# Comparative In Vitro Apoptotic Activity of Two Chimeric Anti-CD99 Antibodies Recognizing Distinct CD99 Regions in T-ALL Models

**DOI:** 10.3390/biomedicines14091913

**Published:** 2026-08-26

**Authors:** Phasinee Juengsamretkarn, Phakhwan Sampaoloi, Kadkanok Ruangkul, Manatchanok Chinakarapong, Praweekorn Pliensak, Myint Myat Thu, Tawan Chokepaichitkool, Supansa Pata, Witida Laopajon, Watchara Kasinrerk, Nuchjira Takheaw

**Affiliations:** 1 Division of Clinical Immunology, Department of Medical Technology, Faculty of Associated Medical Sciences, Chiang Mai University, Chiang Mai 50200, Thailand; 2 Biomedical Technology Research Center, Faculty of Associated Medical Sciences, Chiang Mai University, Chiang Mai 50200, Thailand; 3 Department of Clinical Pathology, Central Chest Institute of Thailand, Nonthaburi 11000, Thailand; 4 Thailand Excellence Center for Tissue Engineering and Stem Cells, Department of Biochemistry, Faculty of Medicine, Chiang Mai University, Chiang Mai 50200, Thailand

**Keywords:** CD99, therapeutic antibody, apoptosis, T-ALL, chimeric antibody

## Abstract

**Background/Objectives:** Therapeutic antibodies have become an important modality for cancer treatment; however, effective targeted antibody therapies for T-cell acute lymphoblastic leukemia (T-ALL) remain limited. CD99 is a promising therapeutic target due to its high expression in leukemic T cells and its capacity to transmit apoptotic signals. However, the efficacy of antibody-mediated apoptosis may depend on the CD99 region recognized by the antibody. This study compared the in vitro apoptotic activity of two chimeric anti-CD99 antibodies, ChAbMT99/1 and ChAbMT99/3, which recognize distinct regions of CD99 in T-ALL models. **Methods:** ChAbMT99/1 and ChAbMT99/3 were generated as human IgG1 antibodies. Antibody reactivity and binding site specificity were characterized using peptide-based ELISA. Binding to native CD99, apoptosis induction in two-dimensional (2D) Jurkat E6.1 and MOLT-4 suspension cultures and three-dimensional (3D) Jurkat E6.1-derived spheroid models, and cytotoxic effects on peripheral blood mononuclear cells (PBMCs) were assessed by flow cytometry. In vitro hematologic effects were assessed using hemagglutination and platelet aggregation assays. **Results:** ChAbMT99/1 and ChAbMT99/3 specifically recognized peptides corresponding to distinct CD99 regions, with peptide-binding EC_50_ values of 0.813 and 0.819 μg/mL, respectively. Both antibodies exhibited binding reactivity to native CD99 on the tested cells. Functionally, both antibodies induced Annexin V/7-AAD-defined cell death in both 2D and 3D T-ALL models compared with controls in a crosslinking-dependent manner. In contrast, both antibodies induced low levels of cell death (<10%) in bulk PBMCs, with no visible hemagglutination or platelet aggregation observed in samples from five selected donors under the stated assay conditions. **Conclusions:** Both chimeric anti-CD99 antibodies demonstrated in vitro apoptotic activity against T-ALL models despite recognizing distinct regions of CD99. These findings provide a rationale for further investigation of their other mechanisms of action to support the future development of CD99-targeted antibody therapy for T-ALL.

## 1. Introduction

Monoclonal antibodies (mAbs) have become one of the most successful classes of targeted therapeutics in modern oncology because of their high target specificity and favorable pharmacokinetic properties [1,2]. Most clinically approved therapeutic antibodies for cancer exert their antitumor effects primarily through immune-mediated mechanisms, including antibody-dependent cellular cytotoxicity (ADCC), complement-dependent cytotoxicity (CDC), and antibody-dependent cellular phagocytosis (ADCP) [3,4]. However, patients with advanced malignancies or those undergoing intensive chemotherapy frequently exhibit compromised or dysfunctional immune effector cell populations, which may substantially reduce the efficacy of effector-dependent antibody therapies [5,6,7]. Consequently, increasing attention has been directed toward the development of antibodies capable of directly inducing tumor cell death independently of host immune effector systems [8,9]. Antibodies that directly trigger apoptosis may therefore provide therapeutic advantages in immunocompromised patients and in tumor microenvironments with limited immune activity.

Despite their therapeutic potential, a major challenge in developing direct apoptosis-inducing therapeutic antibodies is ensuring their safety, particularly in the treatment of hematologic malignancies such as T-cell acute lymphoblastic leukemia (T-ALL) [10,11]. Many antigen targets in T-ALL are shared between malignant T cells and normal hematopoietic cells, thereby increasing the risk of off-target toxicity and treatment-associated immunodeficiency [10,12]. In addition, several antibody-based therapeutics have demonstrated undesirable interactions with blood components, including the induction of hemagglutination and platelet aggregation, which may limit their clinical applicability [13,14,15]. Therefore, the successful clinical translation of therapeutic antibodies requires developing agents that selectively target malignant cells while maintaining a highly favorable hematologic safety profile.

CD99, also known as MIC2 or E2, is a type I transmembrane glycoprotein expressed in a variety of cell types, including cortical thymocytes, leukocytes, several non-hematopoietic tissues, and leukemic T cells [16]. It is involved in diverse cellular processes, including cell adhesion, migration, differentiation, apoptosis, and tumor progression [16,17,18]. Previous studies have demonstrated that ligation of specific CD99 epitopes can rapidly induce programmed cell death in transformed T-cell lines through a caspase-independent signaling pathway, while exerting minimal effects on normal peripheral T cells [19]. These findings suggest that CD99 may function as a unique death receptor and highlight its potential as a therapeutic target for T-cell malignancies. Furthermore, antibody-mediated engagement of CD99 has been shown to induce apoptotic cell death and suppress tumor progression in several malignancies [16]. In Ewing sarcoma, anti-CD99 antibodies promote caspase-independent cell death [20]. Similarly, anti-CD99 antibodies have been reported to reduce cell viability and inhibit colony formation and cell migration [16]. Collectively, these findings support the development of CD99-targeted antibodies as potential therapeutic agents for hematologic malignancies. Consistent with this concept, CD99 has emerged as a promising therapeutic target for T-ALL because of its high expression in leukemic T cells and its ability to trigger apoptotic signaling following antibody-mediated ligation [11,19,21,22]. In agreement with these findings, our previous studies demonstrated that our in-house anti-CD99 mAb clone MT99/3 selectively induces rapid, crosslinking-dependent apoptosis in leukemic T cells while inducing low levels of cell death in normal peripheral blood mononuclear cells (PBMCs) [23,24]. Importantly, this cytotoxic effect is initiated directly by antibody-mediated receptor ligation and does not strictly depend on immune effector functions. These characteristics suggest that CD99 is an attractive target for investigating antibody-mediated apoptotic activity in leukemia T cells.

However, the therapeutic efficacy of antibodies targeting the same antigen may vary considerably depending on their molecular and functional properties. In addition to antigen expression, factors such as downstream signaling properties, binding affinity, and binding site specificity can influence antibody function and therapeutic activity [4,25]. Among these, binding site specificity has emerged as a critical determinant of biological activity. In particular, binding site specificity can substantially affect antibody orientation and receptor clustering on the cell surface. Structural features such as the antibody elbow angle, defined as the relative orientation between the variable and constant domains, play an important role in determining the spatial arrangement of antibody–antigen complexes [4,9,26]. This phenomenon has been demonstrated in anti-CD20 mAbs, where targeting overlapping but distinct regions can result in altered antibody orientation and differential biological activities, including direct cell death induction or complement activation [9,26]. Therefore, exploring distinct regions of CD99 may represent an important strategy for the development of therapeutic antibodies.

This study investigated and compared the in vitro apoptotic activity of two chimeric anti-CD99 antibodies (ChAbs), chimeric antibody clone MT99/1 (ChAbMT99/1) and chimeric antibody clone MT99/3 (ChAbMT99/3). These chimeric antibodies were engineered by fusing the variable domains of parental murine anti-CD99 clones with human IgG1 heavy-chain and kappa light-chain constant regions [27,28]. The binding site specificities of both chimeric anti-CD99 antibodies were characterized using distinct CD99-derived peptides, and their apoptosis-inducing activity, as assessed by Annexin V/7-Aminoactinomycin D (7-AAD) staining, was investigated using both conventional two-dimensional (2D) suspension cultures and three-dimensional (3D) Jurkat E6.1-derived spheroid models. In addition, the effects of the chimeric antibodies on normal PBMCs were evaluated by Annexin V/7-AAD staining, while in vitro hemagglutination and platelet aggregation assays were performed using samples from five selected donors.

## 2. Materials and Methods

### 2.1. Cells and Cell Lines

Jurkat E6.1 and MOLT-4 T-ALL cell lines were obtained from the American Type Culture Collection (ATCC, Manassas, VA, USA). Cells were cultured in Roswell Park Memorial Institute (RPMI) 1640 medium (Gibco, Grand Island, NY, USA, #31800-022) supplemented with 10% heat-inactivated fetal bovine serum (FBS; Gibco, Grand Island, NY, USA, #A5256701) and antibiotics. Cell cultures were maintained at 37 °C in a humidified incubator containing 5% CO_2_. Formal cell-line authentication was not performed during the present study. The cultures were tested for mycoplasma contamination and confirmed to be mycoplasma-negative throughout the study.

Human peripheral blood mononuclear cells (PBMCs) were isolated from fresh heparinized whole blood collected from healthy donors using the standard Ficoll-Hypaque density gradient centrifugation method. Red blood cells (RBCs) and platelets used for indirect immunofluorescence staining were obtained from EDTA-anticoagulated whole blood. For the hemagglutination assay, RBCs were collected from EDTA-anticoagulated blood by centrifugation at 1500× *g* for 5 min. For the platelet aggregation assay, platelet-rich plasma (PRP) was prepared from sodium citrate–anticoagulated blood by low-speed centrifugation at 200× *g* for 20 min. The healthy donors included in this study were 21–35 years of age.

### 2.2. Production and Purification of Chimeric Antibodies MT99/1 and MT99/3 and Human IgG Control Antibody

HEK293T cells stably expressing chimeric antibodies against human CD99 (ChAbMT99/1 and ChAbMT99/3), previously generated as described in [27,28], were cultured in a 75 cm^2^ tissue culture flask containing Dulbecco’s Modified Eagle Medium (DMEM; Gibco, Grand Island, NY, USA, #12800-017) supplemented with 10% FBS and 100 µg/mL zeocin (InvivoGen, San Diego, CA, USA, #ant-zn-1p). Cells were maintained at 37 °C in a humidified 5% CO_2_ incubator. At approximately 80% confluence, cells were washed twice with DMEM to remove residual FBS from the complete culture medium, and the medium was replaced with serum-free 293 SFM II medium (Gibco, Grand Island, NY, USA, #11685-029) with 100 µg/mL zeocin. Culture supernatants were collected after 5–7 days, clarified by high-speed centrifugation, and filtered through a 0.2 µm filter. Antibodies were purified using a HiTrap Protein G column (Cytiva, Marlborough, MA, USA, #17040403, lot: 10275501) on an ÄKTA start purification system (Cytiva, Uppsala, Sweden), followed by buffer exchange into phosphate-buffered saline (PBS) by dialysis. Antibody concentrations were determined by measuring absorbance at 280 nm using a NanoDrop 2000 spectrophotometer (Thermo-Fisher Scientific, Waltham, MA, USA), with nominal concentrations based on total protein.

Human IgG (hIgG), used as an isotype-matched control antibody in subsequent experiments, was purified from human AB serum using the same procedure described above for the chimeric antibodies. Briefly, hIgG was purified using a HiTrap Protein G column (Cytiva, Marlborough, MA, USA, #17040403, lot: 10275501) on an ÄKTA start purification system, followed by buffer exchange into PBS by dialysis. The antibody concentration was determined by measuring the absorbance at 280 nm using a NanoDrop 2000 spectrophotometer, with nominal concentrations based on total protein.

To assess antibody purity and structural integrity following purification, sodium dodecyl sulfate–polyacrylamide gel electrophoresis (SDS-PAGE) and Western blot analysis were performed. SDS-PAGE was conducted using 6% or 10% separating gels under non-reducing and reducing conditions, respectively, with a 4% stacking gel. Dithiothreitol (DTT) was used as a reducing agent under reducing conditions. Protein bands were visualized by Coomassie brilliant blue staining. For Western blot analysis, antibodies separated by SDS-PAGE were transferred onto polyvinylidene fluoride (PVDF) membranes and probed with horseradish peroxidase (HRP)-conjugated polyclonal rabbit anti-human IgA, IgG, IgM, Kappa, Lambda antibody (Dako, Santa Clara, CA, USA, #P0212). Protein bands were visualized using a SuperSignal West Pico Plus Chemiluminescent Substrate (Thermo-Fisher Scientific, Waltham, MA, USA) and detected using a ChemiDoc imaging system (Bio-Rad, Hercules, CA, USA).

To further evaluate antibody purity and the presence of high-molecular-weight aggregates, size exclusion chromatography (SEC)-HPLC was performed using a Superdex 200 Increase 10/300 GL column (Cytiva, Marlborough, MA, USA, #28990944). The column was equilibrated with 0.1 M Tris-HCl (pH 7.2) and operated under isocratic conditions at room temperature with a flow rate of 0.75 mL/min. Protein elution was monitored by ultraviolet absorbance at 210 nm, and chromatograms were analyzed to determine the percentage of high-molecular-weight species (HMWS), intact IgG monomer (purity), and low-molecular-weight species (LMWS) as shown in Figure S1. The antibody concentrations used in subsequent experiments were determined based on the total protein concentration measured by absorbance at 280 nm and were not normalized to the intact IgG monomer fraction determined by SEC-HPLC, which was used only for antibody preparation characterization. In addition, endotoxin levels of the purified chimeric antibodies were determined according to the manufacturer’s instructions using the Pierce LAL Chromogenic Endotoxin Quantitation Kit (Thermo-Fisher Scientific, Waltham, MA, USA, #A39552).

### 2.3. Verification of the Binding Site Specificity of the Purified Chimeric Antibodies Against CD99

The binding site specificity of purified chimeric antibodies was evaluated by enzyme-linked immunosorbent assay (ELISA). Biotinylated CD99-derived peptides corresponding to the binding regions recognized by ChAbMT99/1 (Biotin-Ahx-DGGFDLSDALPDNENKKPTAIPKKPSAG) and ChAbMT99/3 (Biotin-Ahx-VDGENDDPRPP), each containing biotin-6-aminohexanoic acid (Ahx) at the N-terminus, were synthesized by GenScript. A 96-well ELISA plate (Costar, NY, USA, #3590) was coated with 50 µL/well of avidin (10 µg/mL in coating buffer) and incubated overnight at 4 °C in a humidified chamber. After washing with PBS containing 0.05% Tween-20 (PBST), wells were blocked with 100 µL/well of 2% bovine serum albumin (BSA) in PBS at 37 °C for 1 h. Following washing, biotinylated CD99 peptides diluted to 2 µg/mL were added to the wells (50 µL/well) and incubated at 37 °C for 1 h. After washing, a secondary blocking step was performed using 100 µL/well of 2% skim milk in PBS for 30 min at 37 °C, followed by washing with PBST. Purified ChAbMT99/1, ChAbMT99/3, or isotype-matched control antibodies (human IgG) were serially diluted three-fold, starting at 0.5 µg/mL, added to the corresponding wells, and incubated at 37 °C for 1 h. After washing, HRP-conjugated goat anti-human IgG (Fcγ fragment-specific) antibody (Jackson ImmunoResearch, West Grove, PA, USA, #129968), diluted 1:5000, was added and incubated at 37 °C for 1 h. After a final wash step, 3,3′,5,5′-tetramethylbenzidine (TMB) substrate was added for color development, and the reaction was stopped with 1 N HCl. Absorbance was measured at 450 nm using a microplate reader (Hercuvan, London, UK).

### 2.4. Determination of Chimeric Antibody Binding to Native CD99 and CD99 Expression in T-ALL Cell Lines and Normal Blood Cells

ChAbs and hIgG were biotinylated for use in the cell-based binding reactivity assays and assessment of CD99 expression in Jurkat E6.1 and MOLT-4 cells and human PBMCs. Biotinylation was employed because the use of non-biotinylated ChAbs with a secondary anti-human IgG antibody resulted in increased background staining associated with endogenous membrane IgG in bulk PBMCs. Briefly, ChAbs and hIgG were biotinylated using EZ-Link™ Sulfo-NHS-Biotin (Thermo Fisher Scientific, Waltham, MA, USA, #21217) according to the manufacturer’s instructions. The antibodies were incubated with the biotin reagent at a 10-fold molar excess for 30 min at room temperature. Excess free biotin was removed by dialysis against PBS, and the concentrations of the biotinylated antibodies were re-determined by measuring absorbance at 280 nm using a NanoDrop 2000 spectrophotometer. Following biotinylation, flow cytometry was performed to assess whether biotinylation affected the binding activity of the labeled antibodies. The corresponding results are shown in Figure S2.

CD99-positive Jurkat E6.1 and MOLT-4 cells, as well as human PBMCs (1 × 10^5^ cells/condition), were incubated on ice for 30 min with 50 µL of 20% heat-inactivated human AB serum (Sigma-Aldrich, Louis, MO, USA, #H6914) to block Fc receptors or non-specific binding. Subsequently, 50 µL of four-fold serial dilutions of biotinylated purified ChAbMT99/1, ChAbMT99/3, isotype-matched control antibody (human IgG), starting from a final concentration of 40 µg/mL, were added and incubated on ice for 30 min. After washing, cells were incubated on ice for 30 min with FITC-conjugated streptavidin (1:100 dilution; BioLegend, San Diego, CA, USA, #405202, lot: B259880) and PerCP-conjugated mouse anti-human CD14 mAb (2 µL/test; clone M5E2, BioLegend, San Diego, CA, USA, #301848). Stained cells were analyzed using an Accuri C6 plus flow cytometer (BD Biosciences, San Jose, CA, USA), and flow cytometric data were processed using FlowJo software (version 10.10.1, BD Biosciences, San Jose, CA, USA).

For RBC and platelet staining, 2 µL of EDTA-anticoagulated whole blood was incubated with 50 µL of ChAbMT99/1, ChAbMT99/3, or isotype-matched control antibody (human IgG) at a final concentration of 20 µg/mL for 30 min on ice. After washing, cells were stained with Alexa Fluor 488-conjugated goat anti-human IgG (1:250 dilution; Jackson ImmunoResearch, West Grove, PA, USA, #109-545-098) and PE-conjugated anti-human CD42b antibody (5 µL/test; clone HIP1, BioLegend, San Diego, CA, USA, #303906) for 30 min on ice. Stained samples were analyzed using an Accuri C6 flow cytometer (BD Biosciences), and flow cytometric data were processed using FlowJo software (version 10.10.1). The gating strategy for T-ALL cell lines, normal PBMCs, RBC, and platelet staining is shown in Figure S3. The Minimum Information about a Flow Cytometry Experiment (MIFlowCyt) reporting information is provided in Supplementary Table S9.

### 2.5. Establishment of Jurkat E6.1-Derived Spheroid Model

Jurkat E6.1 cells were seeded into non-treated U-bottom 96-well plates (Thermo-Fisher Scientific, Waltham, MA, USA, #262162) at densities of 6000, 8000, or 10,000 cells/well. Cells were cultured in RPMI 1640 medium supplemented with 10% FBS in the presence or absence of 1% methylcellulose (viscosity: 4000 cP; Sigma-Aldrich, Louis, MO, USA, #M0512) to promote cell aggregation and prevent adherence to the plate surface, as previously described by [29]. Cells were incubated at 37 °C in a humidified 5% CO_2_ incubator for 24, 48, or 72 h. Spheroid formation, representing a 3D in vitro tumor model, was monitored and imaged using an inverted light microscope (Carl Zeiss Microscopy, LLC, White Plains, NY, USA) at 100× magnification.

### 2.6. Apoptosis Assay

T-ALL cell lines (Jurkat E6.1 and MOLT-4) and human PBMCs (1 × 10^5^ cells/50 µL) were pretreated with 50 µL of ChAbMT99/1, ChAbMT99/3, isotype-matched control antibody (human IgG), or medium alone (no-antibody control) at final concentrations of 20, 2, and 0.2 μg/mL in V-bottom 96-well plates for 30 min at room temperature. Cells were then centrifuged, and excess antibodies were completely removed. Subsequently, cells were incubated with or without a secondary antibody crosslinker, goat anti-human IgG, whole molecule (Jackson ImmunoResearch, West Grove, PA, USA, #109-005-098, lot: 176785), at a final concentration of 5 μg/mL in a total volume of 150 µL at 37 °C in a humidified 5% CO_2_ incubator for 90 min. After incubation, cells were harvested and stained with Annexin V–FITC (BioLegend, San Diego, CA, USA, #640945, lot: B404229 and B480747) and 7-AAD (0.4 µg/mL; BioLegend, San Diego, CA, USA, #420404, lot: B402850). to assess apoptosis. The percentage of apoptotic cells was analyzed using an Accuri C6 plus flow cytometer (BD Biosciences) and FlowJo software (version 10.10.1).

For the 3D Jurkat E6.1-derived spheroid model, spheroids were generated in non-treated U-bottom 96-well plates at a density of 6000 cells/well and cultured at 37 °C in a humidified 5% CO_2_ incubator for 72 h. Spheroids were washed three times with PBS and pretreated with 50 µL of ChAbMT99/1, ChAbMT99/3, or isotype-matched control antibody at a final concentration of 20 μg/mL. After centrifugation and complete removal of excess antibodies, spheroids were incubated with goat anti-human IgG secondary antibody crosslinker (Jackson ImmunoResearch, West Grove, PA, USA, #109-005-098) at a final concentration of 5 μg/mL in a total volume of 150 µL at 37 °C in 5% CO_2_ incubator for 90 min. Spheroids were then mechanically dissociated into single cells by repeated pipetting using a 200 µL pipette. The dissociated cells were stained with Annexin V–FITC and 7-AAD. The percentage of apoptotic cells was analyzed using an Accuri C6 plus flow cytometer (BD Biosciences) and FlowJo software (version 10.10.1). MIFlowCyt reporting information for the apoptosis assay is provided in Supplementary Table S9.

### 2.7. Hemagglutination Assay

A 2% (*v*/*v*) human RBCs suspension in PBS, prepared from EDTA-anticoagulated blood, was used for the hemagglutination assay. Briefly, 25 µL of the 2% RBC suspension was incubated with 75 µL of ChAbMT99/1, ChAbMT99/3, or isotype-matched control antibody (human IgG) at various concentrations in U-bottom 96-well plates for 60 min at room temperature. Anti-D IgM/IgG antibody (1:2000 dilution; The National Blood Center, Thai Red Cross Society, Bangkok, Thailand) was used as a positive control. Hemagglutination was evaluated visually based on the pattern of RBC sedimentation and lattice formation in the tested wells. Each donor sample was evaluated once.

### 2.8. Platelet Aggregation Assay

Briefly, 80 µL of PRP was incubated with 20 µL of ChAbMT99/1, ChAbMT99/3, or isotype-matched control antibody at various concentrations in a flat-bottom 96-well plate (Costar, NY, USA, #3599) for 30 min at room temperature. Adenosine diphosphate (ADP; 10 µM; HiMedia, Kennett Square, PA, USA, #MB191) was used as a positive control to induce platelet aggregation. Platelet aggregation was evaluated by microscopic observation using an inverted microscope at 400× magnification based on the presence or absence of visible platelet aggregates compared with the untreated control, and at least 10 fields were examined for each condition. Each donor sample was evaluated once.

### 2.9. Statistical Analysis

Data are presented as mean ± standard deviation (SD). Statistical analyses were performed using the Wilcoxon matched-pairs signed rank test and one-way ANOVA, followed by Tukey’s multiple comparisons test in GraphPad Prism version 10 (GraphPad, San Diego, CA, USA). A *p* value < 0.05 was considered statistically significant.

## 3. Results

### 3.1. Purity and Structural Integrity of Purified Chimeric Anti-CD99 Antibodies

Following production and purification, the purification yields of the two chimeric antibodies were determined. Protein G affinity purification yielded 10.07 mg of purified ChAbMT99/1 from 320 mL of culture supernatant and 2.60 mg of purified ChAbMT99/3 from 750 mL of culture supernatant (Table S1).

**Figure 1:**
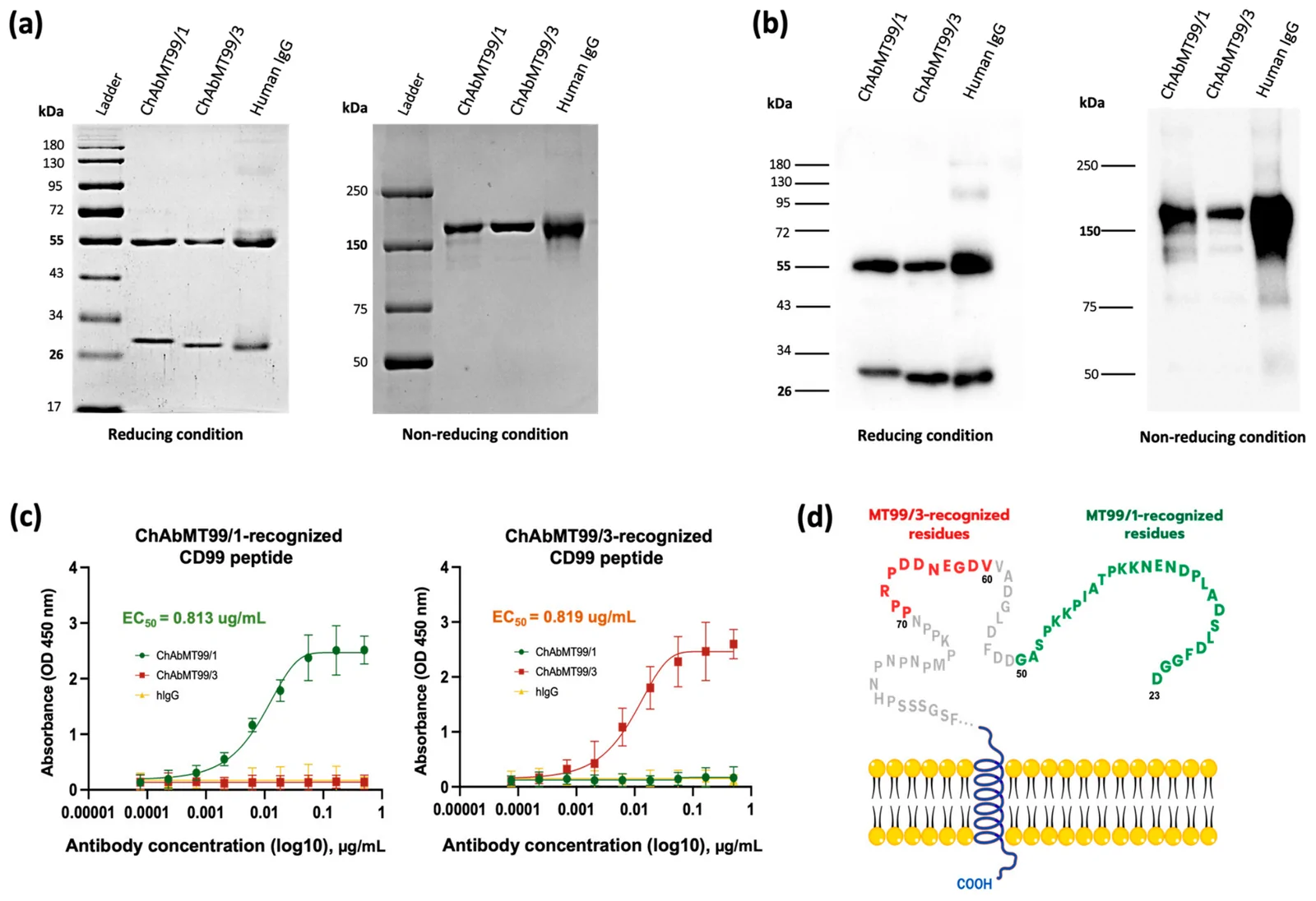
Purity, structural integrity, and binding site specificity of chimeric anti-CD99 antibodies. (**a**) SDS-PAGE analysis of purified chimeric antibodies, ChAbMT99/1 and ChAbMT99/3, under non-reducing (6% separating gel) and reducing (10% separating gel) conditions. Human IgG, used as an isotype-matched control, was included. Gels were stained with Coomassie Brilliant Blue. (**b**) Western blot analysis under reducing and non-reducing conditions. Membranes were probed with HRP-conjugated rabbit anti-human IgA, IgG, IgM, kappa, and lambda antibodies, and signals were detected by chemiluminescence. (**c**) ELISA analysis of the binding site specificity of ChAbMT99/1 and ChAbMT99/3 toward their corresponding CD99-derived peptides. Human IgG (hIgG) was used as an isotype-matched control antibody. Signal was measured as absorbance at 450 nm. Data are presented as mean ± SD from three independent experiments. EC_50_ (half maximal effective concentration) values were calculated using GraphPad Prism. (**d**) Schematic representation of the CD99 extracellular domain showing the amino acid sequence recognized by ChAbMT99/1 (residues 23–50; green) and ChAbMT99/3 (residues 60–70; red).

SDS-PAGE and Western blot analyses were subsequently performed to assess antibody purity and structural integrity. Under reducing conditions, SDS-PAGE analysis of the purified chimeric antibodies and hIgG revealed two major bands corresponding to the heavy chain (HC, ~55 kDa) and light chain (LC). The estimated molecular weights of the LC were 29.2 kDa for ChAbMT99/1, 28.3 kDa for ChAbMT99/3, and 27.8 kDa for hIgG, as determined by ImageJ software version 1.54 relative to a protein molecular weight marker. Under non-reducing conditions, both purified chimeric antibodies and hIgG migrated as major bands with an estimated molecular weight of approximately 180 kDa, consistent with the expected size of intact IgG molecules (Figure 1a). Western blot analysis using anti-human IgA, IgG, IgM, kappa, and lambda antibodies detected major bands corresponding to those observed in the SDS-PAGE analysis and showed patterns similar to those of the hIgG used as an isotype-matched control in this study under both reducing and non-reducing conditions (Figure 1b). These findings demonstrate the successful production and purification of the chimeric IgG antibodies and hIgG. To further characterize antibody quality, SEC-HPLC analysis demonstrated monomeric intact IgG of 73.66% for ChAbMT99/1 and 82.24% for ChAbMT99/3. HMWS accounted for 2.41% and 6.46%, respectively, whereas LMWS accounted for 23.93% and 11.30%, respectively (Figure S1). Endotoxin levels were evaluated using the chromogenic Limulus amebocyte lysate (LAL) assay at the antibody concentrations used in the experiments, with a total treatment volume of 100 µL per condition, and are reported as EU/mL. At 20 μg/mL, the estimated endotoxin level was 0.698 EU/mL for ChAbMT99/1, whereas the value for ChAbMT99/3 was above the upper quantifiable range of the assay (>1.0 EU/mL). At 2 μg/mL, the estimated endotoxin levels were 0.039 and 0.650 EU/mL for ChAbMT99/1 and ChAbMT99/3, respectively. At 0.2 μg/mL, ChAbMT99/3 showed an estimated endotoxin level of 0.040 EU/mL, whereas the ChAbMT99/1 value was below the quantifiable range. Collectively, the SEC-HPLC and endotoxin results support the use of the purified chimeric antibodies for subsequent functional characterization.

### 3.2. Distinct Binding Site Specificity of ChAbMT99/1 and ChAbMT99/3

The binding site specificity of purified ChAbMT99/1 and ChAbMT99/3 was evaluated by ELISA using CD99-derived peptides corresponding to the extracellular regions recognized by each antibody. Both chimeric antibodies exhibited dose-dependent binding to peptides corresponding to distinct regions of CD99, indicating that ChAbMT99/1 and ChAbMT99/3 recognize different regions of CD99 (Figure 1c). The amino acid sequences and positions of these binding regions within the extracellular domain of CD99 are shown in Figure 1d. ChAbMT99/1 showed specific binding to its corresponding CD99 peptide, with a half maximal effective concentration (EC_50_) value of 0.813 µg/mL, whereas ChAbMT99/3 specifically bound to its corresponding CD99 peptide, with an EC_50_ value of 0.819 µg/mL (Figure 1c). Human IgG, used as an isotype-matched control, showed no detectable binding to any coated peptides (Figure 1c). These results demonstrate that both chimeric antibodies specifically recognize their respective CD99 peptide regions. However, binding to synthetic peptides may not fully reflect antibody recognition of native CD99. Therefore, we further evaluated the binding of the chimeric antibodies to native CD99 expressed in T-ALL cell lines and normal blood cells using indirect immunofluorescence staining followed by flow cytometric analysis.

### 3.3. Binding Reactivity of ChAbMT99/1 and ChAbMT99/3 to Native CD99

To evaluate whether biotinylation affected antibody binding, flow cytometry was performed to compare the binding of non-biotinylated and biotinylated antibodies to Jurkat E6.1 cells using anti-human IgG–Alexa Fluor 488 (Figure S2). For human IgG, the geometric mean fluorescence intensity (GeoMFI) of Alexa Fluor 488 was 293 and 247 for the non-biotinylated and biotinylated antibodies, respectively. For ChAbMT99/1, the GeoMFI was 73,937 for the non-biotinylated antibody and 92,198 for the biotinylated antibody, whereas for ChAbMT99/3, the GeoMFI was 68,248 and 67,811, respectively. Overall, the binding signals observed before and after biotinylation indicated that the biotinylation did not substantially affect the binding activity of ChAbMT99/1 or ChAbMT99/3.

**Figure 2:**
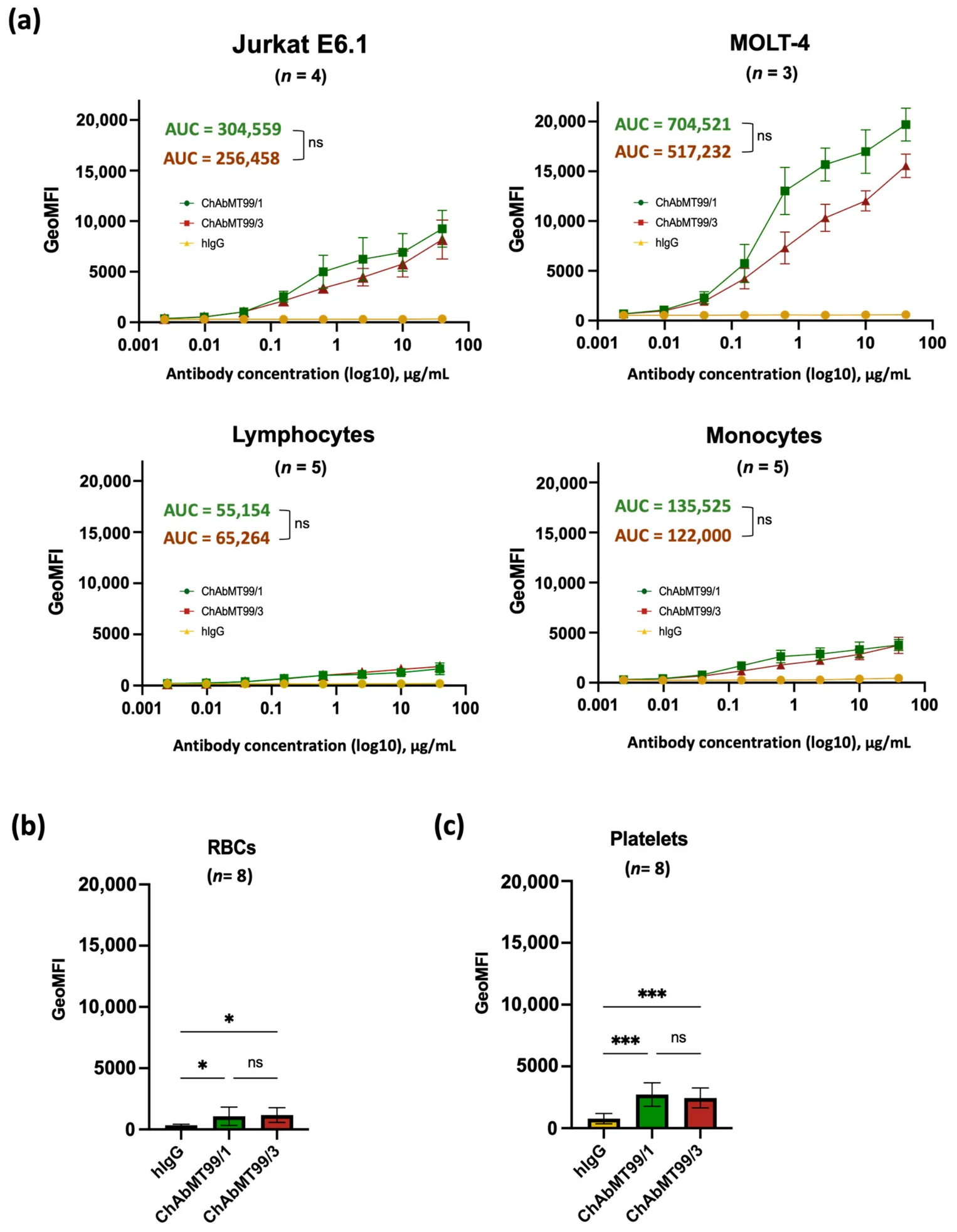
Binding reactivity of chimeric anti-CD99 antibodies and CD99 expression in T-ALL cell lines and normal human blood cells. (**a**) T-ALL cell lines and normal PBMCs, gated as lymphocytes and monocytes, were stained with biotinylated chimeric antibodies (ChAbMT99/1 and ChAbMT99/3) or biotinylated isotype-matched control antibody (hIgG) at various concentrations, followed by detection with streptavidin-FITC. Binding and CD99 expression levels were analyzed by flow cytometry, and the area under the curve (AUC) values were compared using the Wilcoxon matched-pairs signed rank test; ns, non-significant. Exact *p*-values are provided in the Supplementary Materials (Table S2). (**b**,**c**) The binding activity of chimeric anti-CD99 antibodies and CD99 expression in normal RBCs and platelets were evaluated using Alexa Fluor 488-conjugated goat anti-human IgG and analyzed by flow cytometry. Statistical significance was determined using one-way ANOVA followed by Tukey’s multiple comparisons test. * *p* < 0.05, *** *p* < 0.001, and ns, non-significant. Exact *p*-values and 95% confidence intervals (CI) for all pairwise comparisons are provided in the Supplementary Materials (Table S3). (**a**–**c**) Data are presented as mean ± SD. For experiments using Jurkat E6.1 and MOLT-4 cell lines, *n* represents the number of independent biological experiments. For experiments using normal human blood cells (lymphocytes, monocytes, RBCs, and platelets), *n* represents the number of individual healthy donors, as indicated in each graph.

Indirect immunofluorescence staining followed by flow cytometric analysis was performed to evaluate the binding reactivity of the chimeric antibodies to native CD99. T-ALL cell lines (Jurkat E6.1 and MOLT-4) and normal blood cells, including PBMCs, RBCs, and platelets, were examined for their endogenous CD99 expression. To evaluate antibody binding, a range of concentrations of biotinylated antibodies was tested on T-ALL cell lines, lymphocytes, and monocytes. Among all tested cells, the biotinylated human IgG isotype control showed low/background binding, indicating minimal nonspecific cell-surface binding under the assay conditions. Both biotinylated chimeric anti-CD99 antibodies showed concentration-dependent binding, with no statistically significant difference in their area under the curve (AUC) values in these small exploratory datasets (Figure 2a). However, differences in native binding characteristics, including affinity, avidity, epitope accessibility, or labeling efficiency, cannot be completely excluded. For RBCs and platelets, no statistically significant differences in binding were observed between the two chimeric antibodies (Figure 2b,c).

In addition, CD99 expression levels on T-ALL cell lines and normal blood cells were investigated. Based on GeoMFI, CD99 expression was higher in both T-ALL cell lines than in normal blood cells, with MOLT-4 cells exhibiting the highest expression level. These findings showed that both chimeric anti-CD99 antibodies bind to native CD99 on T-ALL cell lines and PBMCs. Therefore, PBMCs from the same donors used in the binding assay were subsequently used in the apoptosis assay.

As shown in Figure 2b,c, CD99 expression profiles on RBCs and platelets were further analyzed according to individual donors, as summarized in Table 1. The percentage of CD99-positive RBCs and platelets was determined after background correction by subtracting values obtained with the isotype-matched control antibody. Notably, two donors (N2, blood group A; and N5, blood group AB) showed minimal CD99-positive reactivity in RBCs (≤1.1%) with both antibodies, whereas the remaining six donors exhibited >20% CD99-positive RBCs, with considerable interindividual variability and no apparent association with ABO blood group. In contrast, platelets from all donors showed CD99-positive binding with both chimeric antibodies, including donors N2 and N5, although the levels varied among individuals.

Because variable percentages of CD99-positive RBCs and platelets were observed among donors for both ChAbMT99/1 and ChAbMT99/3, selected donors representing a broad range of CD99-positive cell percentages were further evaluated in hemagglutination and platelet aggregation assays. For the purpose of donor selection, the percentage of CD99-positive cells was categorized as low (0–20%), intermediate (21–50%), and high (>50%) based on flow cytometric analysis. Donors were selected to represent all major ABO blood groups and a broad range of CD99-positive cell percentages. To provide a stringent assessment of potential antibody-mediated effects on RBCs and platelets, three of the five selected donors exhibited high percentages (>50%) of CD99-positive RBCs and/or platelets. One of the eight donors who provided samples for the RBC and platelet experiments also provided PBMCs for the native CD99-binding and apoptosis assays.

### 3.4. Chimeric Anti-CD99 Antibodies Induce Crosslinking-Dependent Cell Death in T-ALL Cells

**Figure 3:**
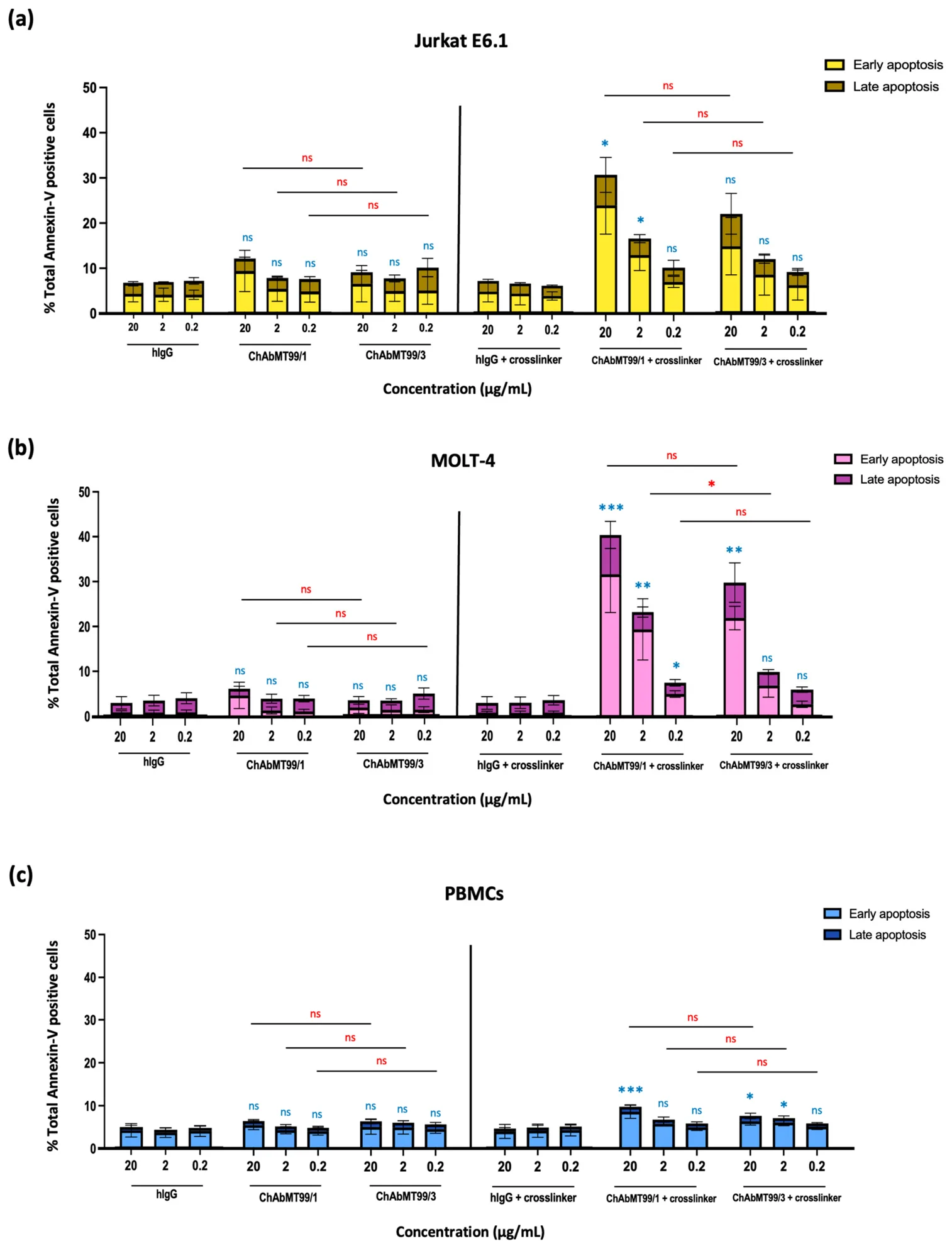
Cell death induced by ChAbMT99/1 and ChAbMT99/3 in T-ALL cell lines compared with normal PBMCs. (**a**) Jurkat E6.1 cells (*n* = 3), (**b**) MOLT-4 cells (*n* = 3), and (**c**) healthy PBMCs (*n* = 5) were treated with various concentrations of chimeric anti-CD99 antibodies or human IgG (hIgG; isotype-matched control), in the presence or absence of a crosslinker (goat anti-human IgG antibody). After 90 min of incubation, apoptosis was assessed by flow cytometry using Annexin V–FITC and 7-AAD staining. The percentage of total Annexin V-positive cells was calculated as the sum of early apoptotic (Annexin V^+^/7-AAD^−^) and late apoptotic (Annexin V^+^/7-AAD^+^) cells. Data are presented as mean ± SD. Statistical significance was analyzed using repeated-measures one-way ANOVA followed by Tukey’s multiple comparisons test. Blue symbols and asterisks (*) indicate significant differences in total Annexin V-positive cells between each ChAb vs. hIgG at the same antibody concentration: ns, not significant; * *p* < 0.05, ** *p* < 0.01, *** *p* < 0.001. Red symbols and asterisks (*) indicate significant differences in total Annexin V-positive cells between ChAbMT99/1 vs. ChAbMT99/3 at the same antibody concentration: ns, not significant; * *p* < 0.05. Exact *p*-values and 95% confidence intervals (CI) for all pairwise comparisons are provided in the Supplementary Materials (Tables S4–S7).

Previous studies have reported that the parental mouse mAb MT99/1 and mAb MT99/3 can directly induce apoptosis in T-ALL cells [23,30]. In the present study, apoptosis assays using Annexin V-FITC and 7-AAD were performed to evaluate and compare the cell death-inducing activity of ChAbMT99/1 and ChAbMT99/3 in two T-ALL cell lines (Jurkat E6.1 and MOLT-4) and in normal PBMCs, in the presence or absence of a secondary antibody crosslinker. In the presence of the crosslinker, both chimeric antibodies induced Annexin V/7-AAD-defined cell death compared with the hIgG control in T-ALL cell lines in a dose-dependent manner, whereas low levels of cell death (<10%) were observed in bulk PBMCs under the tested short-term conditions (Figure 3). At the same antibody concentrations, ChAbMT99/1 showed higher levels of Annexin V-positive cells than ChAbMT99/3 in both Jurkat E6.1 and MOLT-4 cells; however, the differences between the two antibodies were not statistically significant (Figure 3a,b). Representative flow cytometric plots are shown in Figure S4. These findings indicate that both chimeric anti-CD99 antibodies can induce Annexin V/7-AAD-defined cell death in T-ALL cells in the presence of a secondary antibody crosslinker under the tested in vitro conditions. In contrast, in the absence of the secondary antibody crosslinker, neither ChAbMT99/1 nor ChAbMT99/3 induced a statistically significant increase in apoptosis compared with the hIgG control. Together with the low endotoxin levels detected in the purified antibody preparations, these findings are consistent with CD99-related activity and are unlikely to be explained solely by endotoxin. Although statistically significant increases in apoptotic cells were observed in some PBMC treatment groups compared with the hIgG control, the magnitude of these effects remained substantially lower than that observed in T-ALL cell lines (Figure 3c).

Based on the results obtained in the 2D apoptosis assays, Jurkat E6.1 cells were selected as the T-ALL model for subsequent 3D spheroid experiments, consistent with a previously published approach [29]. The highest antibody concentration tested in the 2D assays was used to assess whether the observed apoptotic activity could be maintained under 3D culture conditions.

### 3.5. Establishment and Evaluation of Apoptosis Induced by Chimeric Anti-CD99 Antibodies in a 3D Jurkat-Derived Spheroid Model

Although ChAbMT99/1 and ChAbMT99/3 significantly induced apoptosis in conventional two-dimensional (2D) suspension cultures, leukemic cells in vivo reside within a complex bone marrow microenvironment that supports leukemic cell survival and contributes to resistance against apoptosis and chemotherapy [31]. Three-dimensional (3D) culture systems have been shown to provide more physiologically relevant in vitro models than conventional 2D cultures by better recapitulating cell–cell interactions, microenvironmental conditions, cellular function, and morphology [29,31]. Therefore, a 3D Jurkat E6.1-derived spheroid model was established to evaluate apoptosis induced by ChAbMT99/1 and ChAbMT99/3.

**Figure 4:**
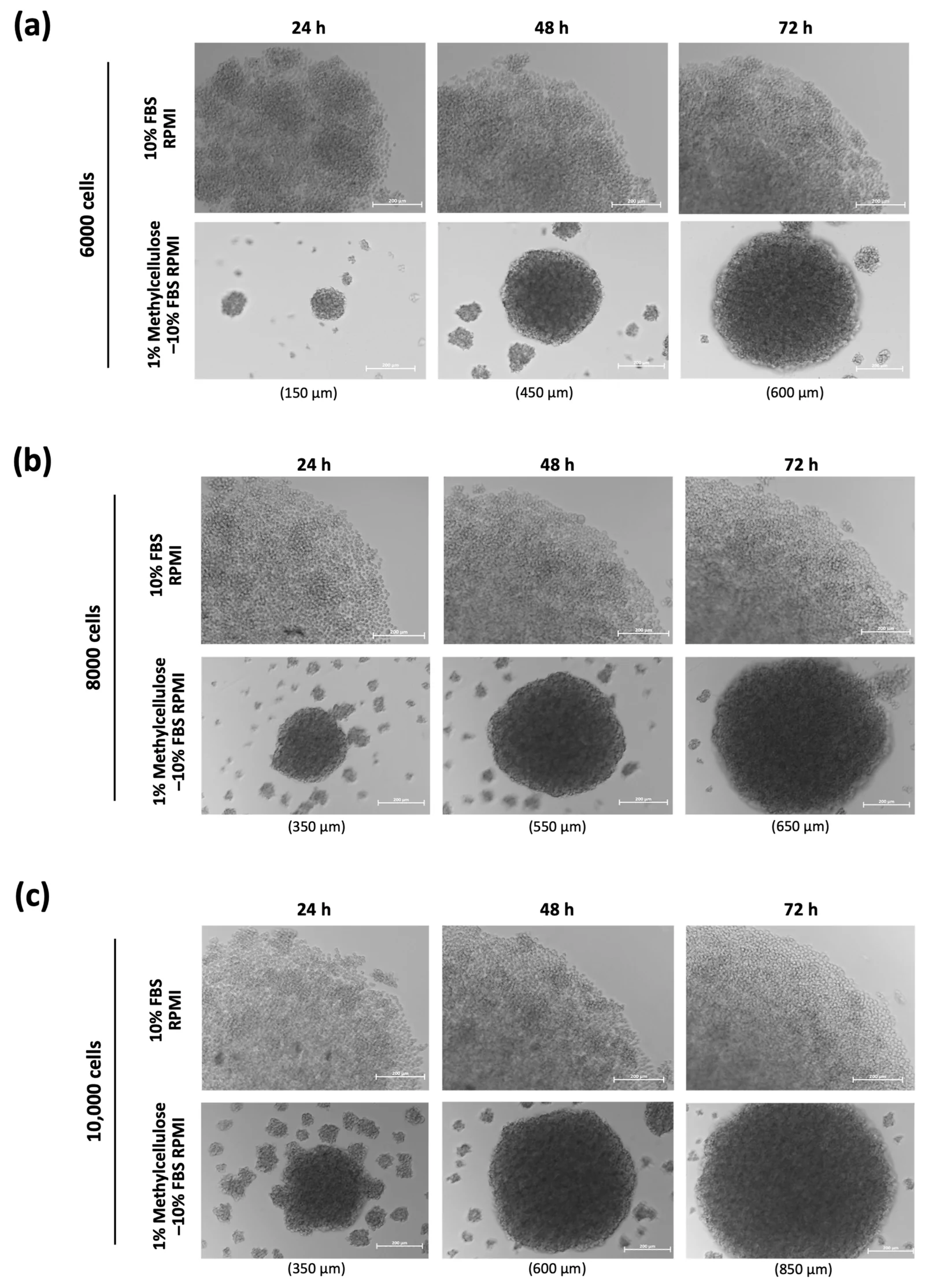
Establishment of a three-dimensional (3D) Jurkat-derived spheroid model. (**a**–**c**) Representative images from two independent experiments. Cells were seeded into non-treated U-bottom 96-well plates at densities of (**a**) 6000, (**b**) 8000, and (**c**) 10,000 cells/well and cultured in 10% FBS-RPMI, in the presence (lower panels) or absence (upper panels) of 1% methylcellulose for 24, 48, and 72 h. Images were acquired using an inverted microscope at 100× magnification. Spheroid diameter (µm), indicated below each image, was measured from the largest spheroid located at the center of each well. Scale bar = 200 µm.

In this study, Jurkat E6.1-derived spheroids were generated and optimized as a 3D in vitro model for subsequent apoptosis assays. Cells were seeded at densities of 6000, 8000, and 10,000 cells/well (Figure 4) in RPMI 1640 medium supplemented with 10% FBS, in the presence or absence of 1% methylcellulose, and incubated for 24, 48, and 72 h. Under inverted microscopy, cells cultured in 10% FBS-RPMI alone remained predominantly single-cell suspensions without spheroid formation (Figure 4; upper panels). In contrast, the presence of 1% methylcellulose promoted cell aggregation and spheroid formation across all seeding densities (Figure 4; lower panels). At 24 h, initial cell clustering was observed across all methylcellulose-treated conditions, with spheroid size increasing in proportion to the initial seeding density. At 48 and 72 h, spheroids became more compact and exhibited increased diameters under all conditions (Figure 4).

Based on these observations, spheroids generated from 6000 cells/well cultured for 72 h in the presence of 1% methylcellulose were selected as the optimal condition for the subsequent apoptosis assay. This condition produced a single compact spheroid with an approximate diameter of 500–600 µm and fewer surrounding satellite aggregates compared with other conditions.

**Figure 5:**
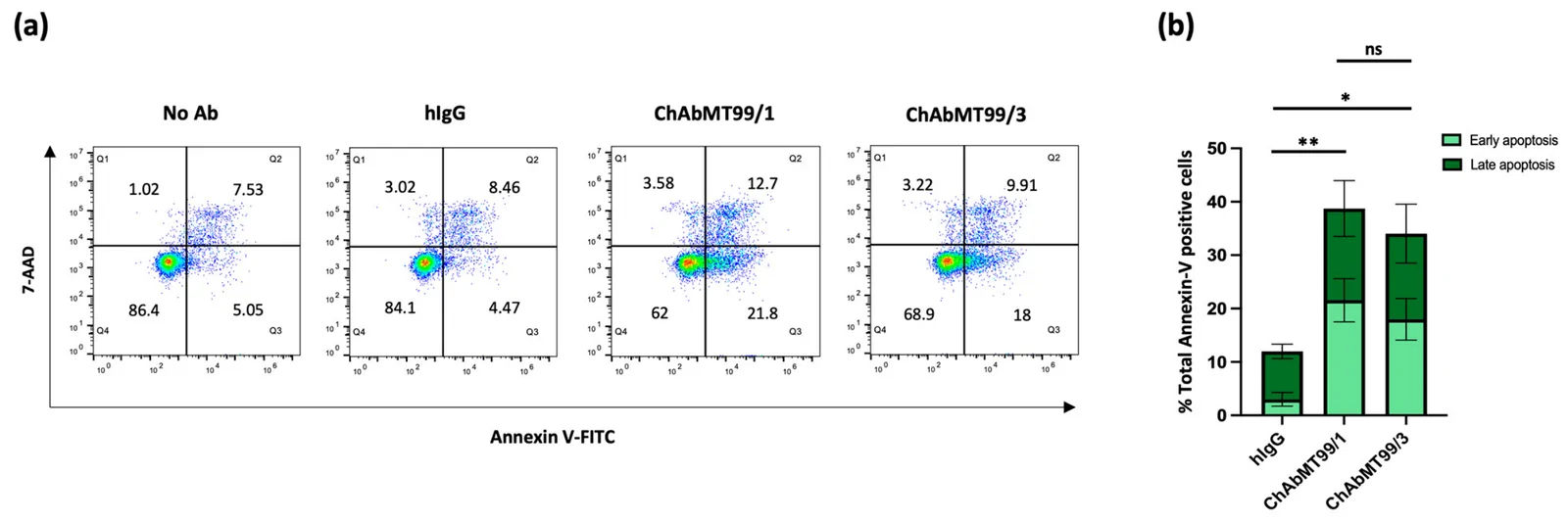
Apoptosis-inducing activity of chimeric anti-CD99 antibodies in a 3D Jurkat-derived spheroid model. (**a**) Representative flow cytometry plots showing apoptosis induced by ChAbMT99/1 and ChAbMT99/3 assessed by flow cytometry using Annexin V–FITC and 7-AAD staining in 3D Jurkat-derived spheroids. Human IgG (hIgG) was used as an isotype-matched control antibody. The percentage of total Annexin V-positive cells was calculated as the sum of early apoptotic (Annexin V^+^/7-AAD^−^) and late apoptotic (Annexin V^+^/7-AAD^+^) cells. The gating strategy is shown in Supplementary Figure S5. (**b**) Bar graphs show the percentage of total Annexin V-positive cells following treatment of Jurkat E6.1-derived spheroids with chimeric anti-CD99 antibodies or the isotype-matched control. Data are presented as mean ± SD from three independent experiments. Statistical significance was analyzed using repeated-measures one-way ANOVA followed by Tukey’s multiple comparisons test. Statistical significance of total Annexin V-positive cells was determined between the groups connected by the corresponding comparison lines. Significance levels are indicated as follows: ns, not significant; * *p* < 0.05, ** *p* < 0.01. Exact *p*-values and 95% confidence intervals (CI) for all pairwise comparisons are provided in the Supplementary Materials (Table S8).

Using the optimized 3D culture condition, Jurkat E6.1-derived spheroids were treated with chimeric antibodies or hIgG isotype-matched control antibody in the presence of a secondary antibody crosslinker for 90 min. Both chimeric anti-CD99 antibodies induced Annexin V/7-AAD-defined cell death compared with the hIgG control in this single Jurkat-derived spheroid model, whereas no statistically significant difference was observed between ChAbMT99/1 and ChAbMT99/3 (Figure 5).

### 3.6. In Vitro Evaluation of the Hematologic Effects of ChAbMT99/1 and ChAbMT99/3

Five donors were selected for hemagglutination and platelet aggregation assays, including N2 (blood group A), N4 (blood group B), N6 (blood group AB), N7 (blood group O), and N8 (blood group O). These donors were selected to represent all major ABO blood groups and a range of CD99 expression levels on RBCs and platelets, including low, intermediate, and high expression profiles.

In the hemagglutination assay, no RBC agglutination was observed with either chimeric anti-CD99 antibodies or isotype-matched control (human IgG) at any tested concentration across all donors (Table 1 and Figure S6), consistent with the negative control wells. In contrast, anti-D IgM/IgG (positive control) showed clear hemagglutination characterized by lattice formation of RBCs. Similarly, in the platelet aggregation assay, no platelet aggregation was detected with either chimeric antibodies or human IgG under inverted microscopy, consistent with the negative control wells (Table 1 and Figure S7). In contrast, treatment with ADP (positive control) induced marked platelet aggregation. These findings provide preliminary evidence of favorable in vitro hematologic compatibility of the chimeric anti-CD99 antibodies.

## 4. Discussion

T-ALL is an aggressive hematologic malignancy that still requires safer and more effective targeted immunotherapies [32,33]. CD99 is highly expressed in leukemic T cells and has been shown to mediate apoptotic signaling following antibody engagement [11,19,21]. The parental mouse monoclonal antibodies MT99/1 and MT99/3 were originally generated by hybridoma technology at our research center and were previously shown to directly induce apoptosis in T-ALL cells [23,30,34]. However, their murine origin may limit clinical application because of potential immunogenicity. To overcome this limitation, the antibodies were further engineered into chimeric forms by fusing murine variable regions with human constant regions, generating ChAbMT99/1 and ChAbMT99/3 of the human IgG1 isotype [27,28]. The epitope recognized by ChAbMT99/3 was identified as amino acid residues 60–70 (VDGENDDPRPP) [23,27]. In contrast, the MT99/3 epitope peptide was not recognized by ChAbMT99/1 in our screening assay, suggesting that the two antibodies recognize distinct extracellular regions of CD99. In the present study, we characterized the binding site specificities of two chimeric anti-CD99 antibodies and compared the in vitro apoptotic activity of both chimeric anti-CD99 antibodies in T-ALL models.

Both ChAbMT99/1 and ChAbMT99/3 were successfully produced in HEK293T cells as structurally intact IgG antibodies and retained specific reactivity toward their corresponding CD99-derived peptides. Both antibodies exhibited their binding reactivity toward native CD99 expressed in leukemic T cells and normal blood cells. Functionally, both chimeric antibodies induced Annexin V/7-AAD-defined apoptotic cell death in T-ALL cell lines in a crosslinking-dependent manner under the present in vitro experimental conditions, while inducing low levels of cell death in bulk PBMCs under the tested short-term conditions. In therapeutic settings, receptor crosslinking may occur through interactions between the Fc portion of the antibody and Fcγ receptors expressed in immune effector cells or endothelial cells, as reported for therapeutic antibodies such as daratumumab and rituximab [35,36]. One limitation of this study is that receptor crosslinking was induced using a goat anti-human IgG secondary antibody, which served as an experimental tool rather than a physiological mediator of antibody clustering. Therefore, future studies using FcγR-expressing effector cells and animal models will be necessary to determine the physiological relevance of this mechanism and to evaluate the therapeutic activity of these chimeric anti-CD99 antibodies under more clinically relevant conditions. Our findings are also consistent with those reported by Romero et al., who demonstrated that efficient CD99-mediated cytotoxicity depends on antibody valency, with constructs possessing a valency of at least three exhibiting significantly greater antitumor activity than lower-valency formats [37]. Together with our observations, these findings suggest that effective CD99-mediated cell death likely depends on multiple factors, including receptor clustering, antibody valency, and the specific binding region.

In this study, the apoptosis assay was performed at 90 min of antibody treatment because CD99-mediated apoptosis occurs rapidly following receptor crosslinking. In our previous study, apoptosis was evaluated at 0, 90, and 180 min after treatment with an anti-CD99 antibody and crosslinker, with the highest proportion of apoptotic cells observed at 90 min, whereas a lower percentage was detected at 180 min [23]. These findings suggest that CD99-induced apoptosis is an early event and support the selection of the 90 min incubation period used in the present study.

Binding site specificity has been reported to critically influence antibody function, as antibodies targeting different binding sites on the same antigen can exhibit distinct biological activities and therapeutic effects [4,9,26]. For example, Rituximab and Ofatumumab, two anti-CD20 antibodies, recognize different extracellular regions of CD20 and consequently exhibit distinct functional properties, including differences in complement activation and the induction of direct cell death [26]. Differences in binding site localization on CD99 may influence the ability of antibody-bound CD99 molecules to form signaling-active clusters required for efficient apoptosis induction. Structural features associated with binding site specificity, including antibody orientation and spatial arrangement following antigen binding, may also contribute to these functional differences [9,26]. Previous studies have demonstrated that different anti-CD99 mAbs induce apoptosis through distinct downstream signaling pathways, including both caspase-dependent and caspase-independent mechanisms [19,38]. In the present study, no statistically significant difference in apoptotic cell death was observed between ChAbMT99/1 and ChAbMT99/3 in T-ALL models under either 2D or 3D culture conditions, despite ChAbMT99/1 showing a higher percentage of total Annexin V-positive cells than ChAbMT99/3. These findings suggest that both chimeric antibodies, which recognize distinct regions of CD99, have potential for further investigation and development as CD99-targeted antibody therapies for T-ALL.

Drug resistance remains a major challenge in the treatment of T-ALL, largely because of the protective effects of the tumor microenvironment [29,39]. Previous studies have shown that Jurkat cells that are sensitive to treatment under conventional 2D culture conditions may exhibit increased resistance in 3D models, which more closely recapitulate the in vivo microenvironment of leukemic cells residing within bone marrow and extramedullary niches [29,31,40]. Therefore, to evaluate therapeutic potential under more physiologically relevant conditions, a 3D Jurkat-derived spheroid model was established and compared with conventional 2D cultures. Importantly, both chimeric anti-CD99 antibodies retained their apoptosis-inducing activity in Jurkat spheroids, supporting the therapeutic potential of anti-CD99 antibodies in T-ALL.

Because CD99 is also expressed in normal hematologic cells, evaluating the hematologic effects of anti-CD99 antibodies is important for the development of CD99-targeted therapies. In this study, neither ChAbMT99/1 nor ChAbMT99/3 induced hemagglutination or platelet aggregation under the tested in vitro conditions in five selected donors, despite detectable binding to RBCs and platelets in some donors. These findings suggest that antibody binding to blood cells expressing relatively lower levels of CD99 than T-ALL cells was insufficient to induce visible hemagglutination or platelet aggregation under the tested in vitro conditions. However, these assays represent only an initial assessment of hematologic compatibility and do not constitute a comprehensive safety evaluation. Additional preclinical studies are required to further characterize the safety profile of these antibodies, including assessments of quantitative hemolysis, complement activation, platelet activation, cytokine release, tissue cross-reactivity, and potential off-target effects in additional normal cell populations before clinical translation.

The present findings also have important implications for the future development of CD99-targeted antibody therapeutics. Other mechanisms of action, including ADCC, ADCP, and CDC, warrant further investigation. Furthermore, optimization of the Fc domain through Fc engineering or glycoengineering may enhance interactions with Fcγ receptor-expressing immune effector cells, thereby improving receptor crosslinking and augmenting both direct apoptotic activity and Fc-mediated effector functions. Alternative antibody formats, including multivalent antibodies, may further enhance receptor clustering and antitumor efficacy. Recent advances in targeted biologics, including Fc-based conjugate engineering, further highlight the potential of antibody optimization strategies to improve therapeutic performance [41].

In parallel, advances in cancer immunotherapy, including adoptive cell therapies such as tumor-infiltrating lymphocyte (TIL) therapy, have highlighted the potential of immune-based approaches to improve clinical outcomes in selected malignancies [42]. Together, these developments underscore the need to develop diverse immunotherapeutic strategies tailored to different cancer types. In this context, our findings suggest that both chimeric anti-CD99 antibodies have potential as candidates for further investigation as CD99-targeted therapeutics for T-ALL.

The limitations of the present study should be acknowledged. For the binding assays using biotinylated antibodies in T-ALL cells and PBMCs, the degree of biotinylation was not quantitatively determined for the chimeric antibodies or the human IgG isotype-matched control. Although flow-cytometric comparison before and after biotinylation did not indicate a substantial loss of antibody binding activity under the tested conditions, differences in labeling efficiency between preparations cannot be completely excluded and should be considered when interpreting the native CD99-binding results. Although apoptosis was primarily assessed by Annexin V/7-AAD staining, which is widely accepted for detecting apoptotic cell death, it does not establish the underlying molecular mechanism; therefore, further mechanistic studies using orthogonal assays are warranted. In addition, a well-established positive apoptosis control, such as staurosporine or etoposide, was not included in this study. Incorporating a validated positive control in future studies would provide additional confirmation of the assay’s responsiveness across experimental runs. Furthermore, spheroid size, hypoxic gradients, drug resistance, antibody penetration, necrosis, and spheroid-to-spheroid variability have been reported to influence therapeutic responses in multicellular tumor spheroids [43]. These factors were not evaluated in the present study and should be considered in future investigations to further characterize antibody activity within three-dimensional tumor models. Another important limitation of this study is that the comparative functional analyses were performed using nominal antibody concentrations based on total protein measured by absorbance at 280 nm rather than concentrations normalized to intact IgG monomer. Although SEC-HPLC was used to characterize the molecular profiles of the purified antibody preparations, the antibodies were not repurified to achieve closely matched monomeric purity before the functional experiments. Therefore, potential differences in molecular composition cannot be completely excluded as contributing to the observed binding and apoptotic responses of the two chimeric antibodies. Thus, the current findings should be considered preliminary and are insufficient to establish a definitive relationship between binding-site localization and comparative functional potency. Future studies using antibodies with closely matched monomeric purity and repeat native-binding and apoptosis analyses will be important to further define the contribution of the CD99-binding region to antibody-mediated activity. Moreover, the human IgG isotype-matched control was characterized by SDS-PAGE and Western blot analysis but was not further evaluated by SEC-HPLC or endotoxin testing. Therefore, differences in molecular composition or endotoxin content between the chimeric antibodies and the isotype control cannot be completely excluded. Nevertheless, the observed crosslinking-dependent apoptotic activity of both chimeric antibodies, together with the low endotoxin levels detected in the chimeric antibody preparations, provides supportive evidence that the observed effects were associated with CD99-related activity rather than being solely attributable to endotoxin. Finally, the assessment of hematologic effects was based on a relatively small number of healthy donors, and platelet aggregation was evaluated qualitatively rather than quantitatively. Future studies incorporating additional T-ALL cell lines, advanced 3D models, patient-derived samples, larger cohorts of healthy donors, Fcγ receptor-mediated activity, in vivo validation, quantitative platelet function assays, and mechanistic investigations will further strengthen the preclinical evaluation of these antibodies and support their further development as CD99-targeted therapeutics.

## 5. Conclusions

Overall, both chimeric anti-CD99 antibodies, which recognize distinct regions of CD99, demonstrated in vitro apoptotic activity against T-ALL models. Although these findings are preliminary, they support further investigation of both antibodies as potential CD99-targeted therapeutics for T-ALL.

## Figures and Tables

**Table 1:** Percentage of CD99-positive cells on RBCs and platelets from eight donors and the effects of chimeric anti-CD99 antibodies on hemagglutination and platelet aggregation.

Donor		CD99-Positive RBCs (%) *		Hemagglutination Results		CD99-Positive Platelets (%) *		Platelet Aggregation Results	
		ChAb MT99/1	ChAb MT99/3	ChAb MT99/1	ChAb MT99/3	ChAb MT99/1	ChAb MT99/3	ChAb MT99/1	ChAb MT99/3
Group A	N1	33.7	48.0	ND	ND	54.3	50.3	ND	ND
	N2	0.0	1.1	Neg	Neg	22.0	12.5	Neg	Neg
Group B	N3	26.3	43.8	ND	ND	57.9	62.4	ND	ND
	N4	20.9	30.8	Neg	Neg	70.9	62.7	Neg	Neg
Group AB	N5	0.1	1.1	ND	ND	46.0	47.1	ND	ND
	N6	53.1	57.5	Neg	Neg	87.0	74.5	Neg	Neg
Group O	N7	55.7	57.5	Neg	Neg	9.9	5.3	Neg	Neg
	N8	73.7	81.8	Neg	Neg	57.8	30.7	Neg	Neg

ND = not done, Neg = negative. * %CD99-positive = (%CD99-positive of tested antibody) − (%CD99-positive of isotype-matched control).

## Data Availability

The data supporting the findings of this study are available within the article and its online Supplementary Materials. Additional data, including raw flow cytometry (.FCS) files, FlowJo workspace (.wsp) files, numerical source data for all graphs, and other supporting datasets, are available from the corresponding author upon reasonable request.

## Supplementary Materials

The following supporting information can be downloaded at: https://www.mdpi.com/article/10.3390/biomedicines14091913/s1.
