# Supplementary material for: Comparative In Vitro Apoptotic Activity of Two Chimeric Anti-CD99 Antibodies Recognizing Distinct CD99 Regions in T-ALL Models
Source: Biomedicines. 2026 Aug 26;14(9):1913. doi: 10.3390/biomedicines14091913 (PMC13604740; doi:10.3390/biomedicines14091913)
Supplement: Supplementary file 1 [file biomedicines-14-01913-s001.zip › biomedicines-4426109-supplementary.pdf]

Supplementary Materials

Comparative In Vitro Apoptotic Activity of Two Chimeric Anti-CD99 Antibodies Recognizing Distinct CD99 Regions in T-ALL Models

Phasinee Juengsamretkarn[1,2], Phakhwan Sampaoloi[1], Kadkanok Ruangkul[1,3], Manatchanok Chinakarapong[2], Praweekorn Pliensak[2], Myint Myat Thu[2], Tawan Chokepaichitkool[4], Supansa Pata[1,2], Witida Laopajon[1,2], Watchara Kasinrerk[2], Nuchjira Takheaw[1,2,*]

(a)

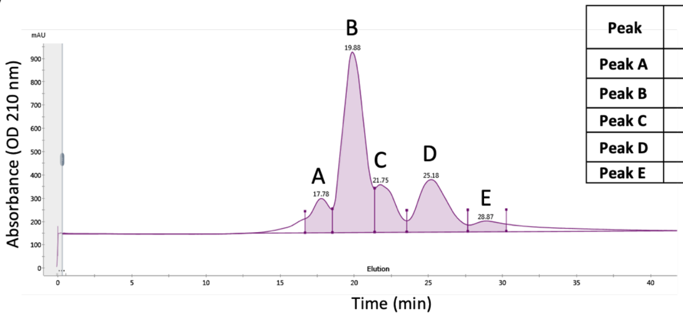

Peak	Retention time (min)	Mw (kDa)	Name
Peak A	17.78	158	Aldolase
Peak B	19.88	75	Conalbumin
Peak C	21.75	44	Ovalbumin
Peak D	25.18	29	Carbonic anhydrase
Peak E	28.87	14	Ribonuclease A

(b)

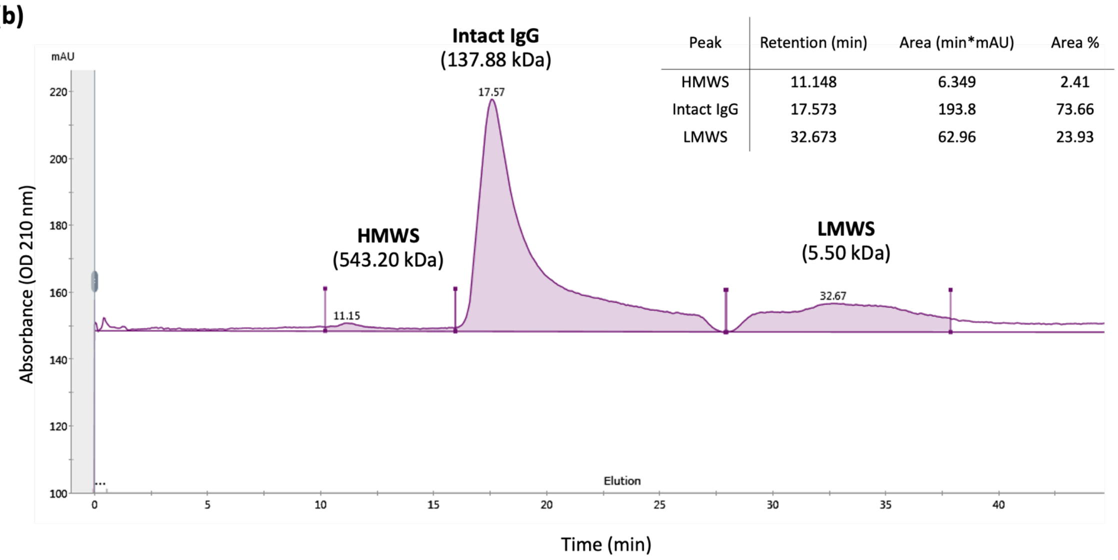

Peak	Retention (min)	Area (min*mAU)	Area %
HMWS	11.148	6.349	2.41
Intact IgG	17.573	193.8	73.66
LMWS	32.673	62.96	23.93

(C)

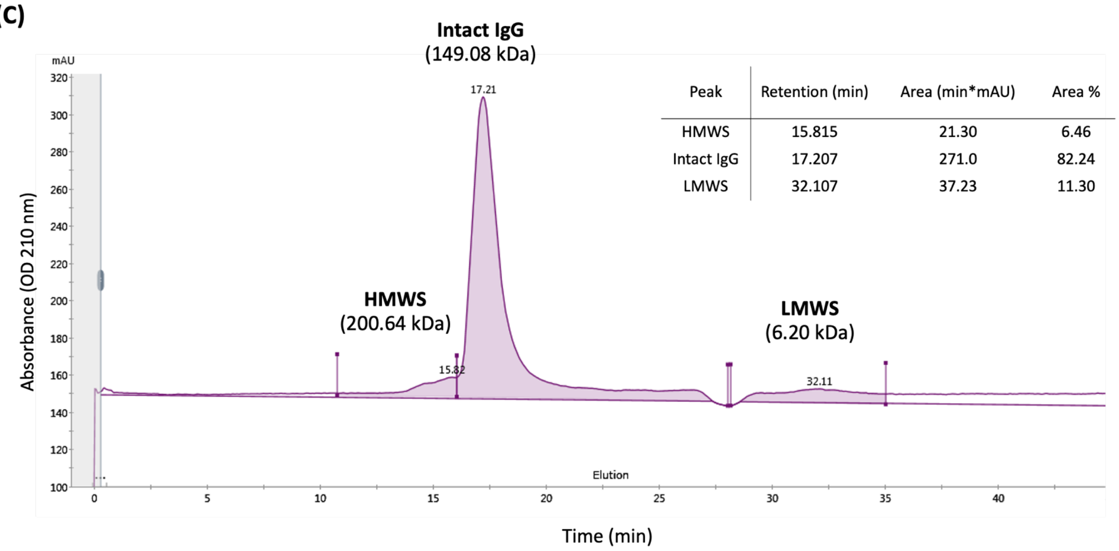

Peak	Retention (min)	Area (min*mAU)	Area %
HMWS	15.815	21.30	6.46
Intact IgG	17.207	271.0	82.24
LMWS	32.107	37.23	11.30

Figure S1. Size-exclusion chromatograms (SEC-HPLC) of chimeric anti-CD99 antibodies. **(a)** SEC-HPLC chromatogram of the standard protein mixture (Cytiva® Gel Filtration Calibration Kit) used for molecular weight estimation. **(b–c)** SEC-HPLC chromatograms of 100 µg of **(b)** ChAbMT99/1 and **(c)** ChAbMT99/3 analyzed using a Superdex 200 Increase 10/300 GL column with 0.1 M Tris-HCl (pH 7.2) as the mobile phase at a flow rate of 0.75 mL/min at room temperature. Peak assignments are high-molecular-weight species (HMWS), intact IgG monomer, and low-molecular-weight species (LMWS), monitored at an absorbance of 210 nm.

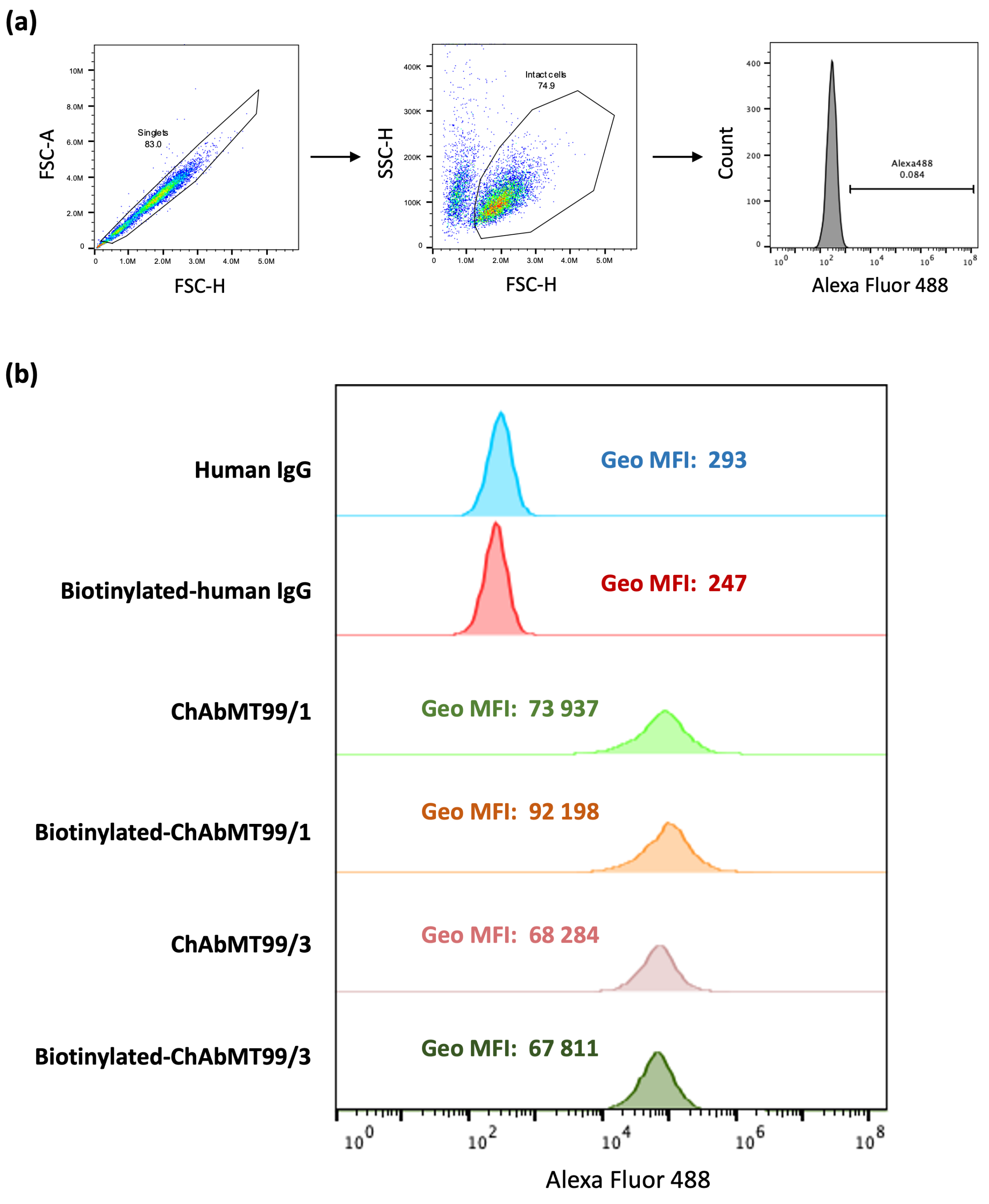

Figure S2. Flow-cytometric evaluation of the effect of biotinylation on antibody binding. **(a)** Gating strategy for flow-cytometric analysis. Singlet cells were first identified based on forward scatter height (FSC-H) and forward scatter area (FSC-A). The singlet population was subsequently gated based on FSC-H and side scatter height (SSC-H) to identify intact cells. The geometric mean fluorescence intensity (GeoMFI) of Alexa Fluor 488 was then determined from

the fluorescence histogram. **(b)** Comparison of antibody binding before and after biotinylation. Jurkat E6.1 cells were stained with non-biotinylated or biotinylated antibodies at a final concentration of 40 μg/mL, followed by detection using goat anti-human IgG–Alexa Fluor 488.

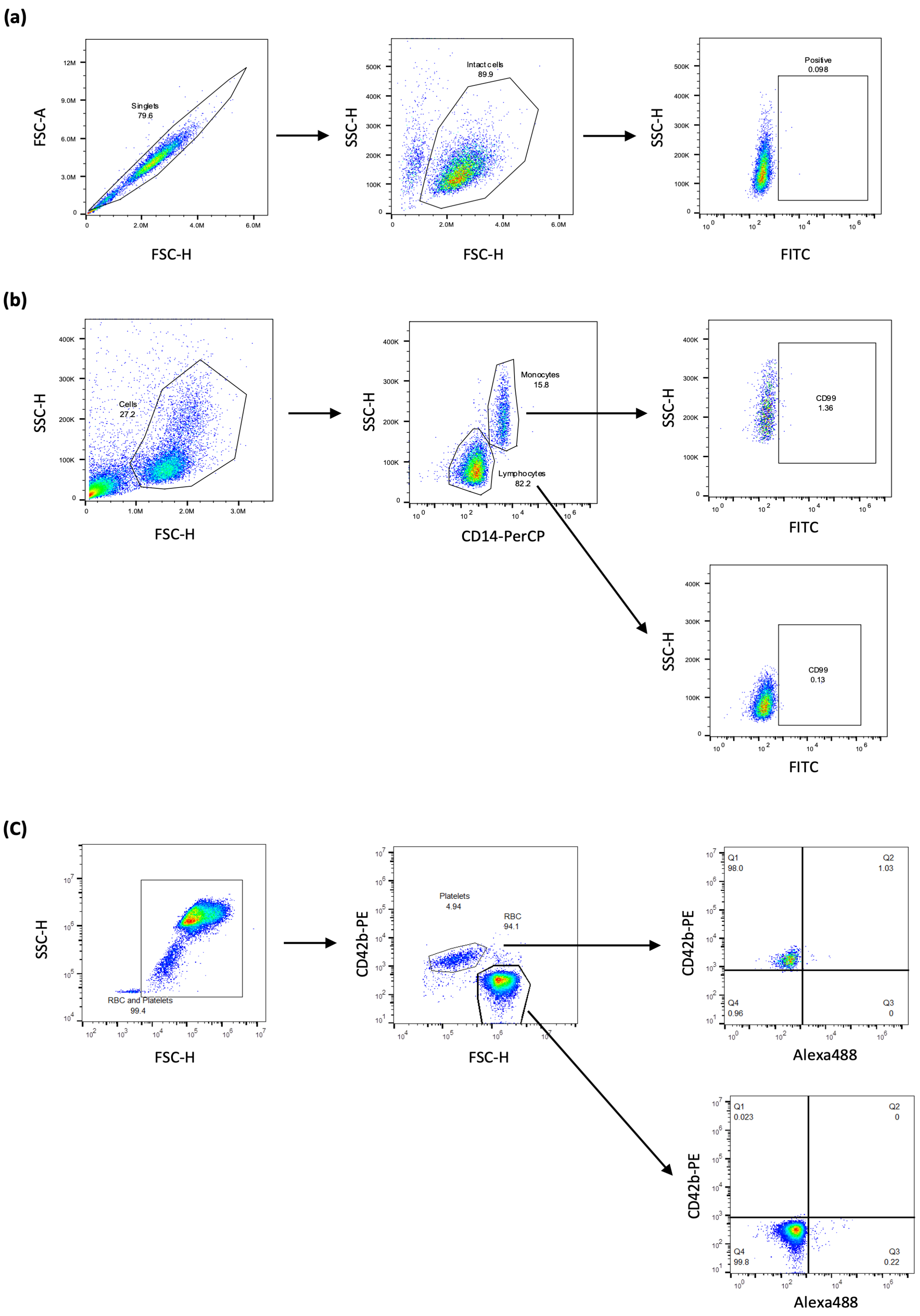

Figure S3. Gating strategy for evaluating the binding reactivity of chimeric anti-CD99 antibodies and CD99 expression in T-ALL cell lines and normal human blood cells by indirect immunofluorescence staining. **(a)** T-ALL cell lines and **(b)** normal PBMCs were stained with

various concentrations of biotinylated chimeric antibodies (ChAbMT99/1 and ChAbMT99/3) or an isotype-matched control antibody (hIgG), followed by detection with streptavidin-FITC. For **(a)**, singlet cells were first identified based on forward scatter height (FSC-H) and forward scatter area (FSC-A). The singlet population was then gated using FSC-H and side scatter height (SSC-H) to identify the cell population. Finally, the geometric mean fluorescence intensity (GeoMFI) of FITC was determined. For **(b)**, PBMCs were initially gated based on FSC-H and SSC-H. The gated cell population was further separated into monocytes (CD14-positive) and lymphocytes by plotting PerCP fluorescence against SSC-H. The GeoMFI of FITC was then determined for each monocyte and lymphocyte population. **(c)** RBCs and platelets were stained with ChAbMT99/1, ChAbMT99/3, or the hIgG control, followed by Alexa Fluor 488-conjugated goat anti-human IgG and PE-conjugated anti-human CD42b antibody as a platelet marker, and analyzed by flow cytometry. RBC and platelet populations were initially gated based on FSC-H and SSC-H using logarithmic scales. The gated cells were further separated into platelets (CD42b-positive) and RBCs by plotting PE fluorescence against FSC-H. Finally, the percentage of CD99-positive cells and the GeoMFI of Alexa Fluor 488 were determined for both RBCs and platelets by plotting PE fluorescence against Alexa Fluor 488.

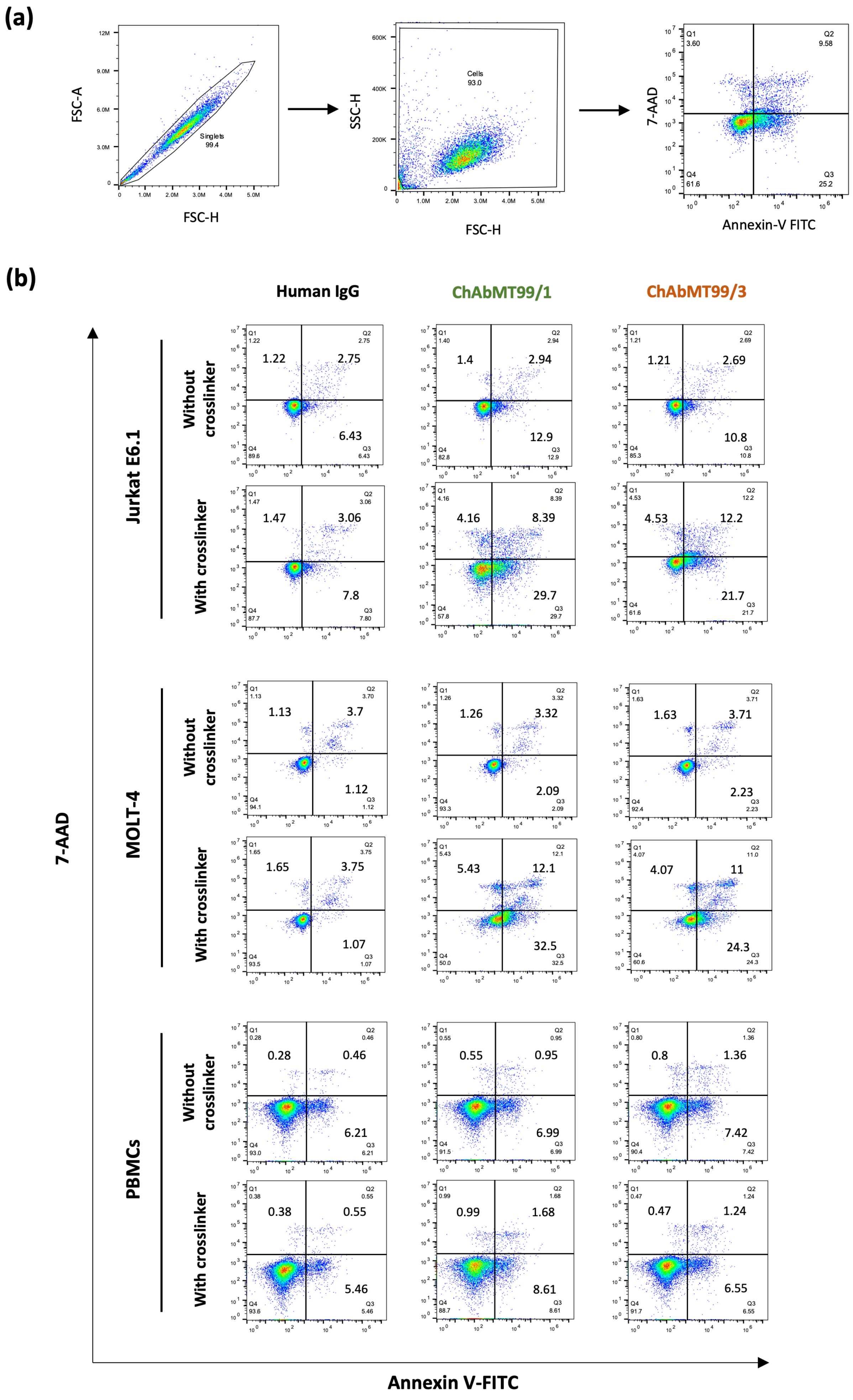

(a)
FSC-A
FSC-H
Singlets 99.4
SSC-H
Cells 93.0
7-AAD
Annexin-V FITC
Q1 3.60
Q2 9.58
Q3 25.2
Q4 61.6
(b)
Human IgG
ChAbMT99/1
ChAbMT99/3
7-AAD
Jurkat E6.1
MOLT-4
PBMCs
Without crosslinker
With crosslinker
Annexin V-FITC

Figure S4. Representative flow cytometric data of the apoptosis assay. **(a)** Gating strategy for apoptosis analysis. Singlet cells were first identified based on forward scatter height (FSC-H) and forward scatter area (FSC-A). The singlet population was subsequently gated based on FSC-H and side scatter height (SSC-H) to identify the cell population. The gated cells were then analyzed using Annexin V–FITC and 7-AAD staining to determine the percentages of live cells (Annexin V–FITC^{-}, 7-AAD^{-}), early apoptotic cells (Annexin V–FITC^{+}, 7-AAD^{-}), late apoptotic cells (Annexin V–FITC^{+}, 7-AAD^{+}), and necrotic cells (Annexin V–FITC^{-}, 7-AAD^{+}). **(b)** Representative flow cytometry plots showing apoptosis in Jurkat E6.1, MOLT-4, and PBMCs after 90 min of treatment, with or without crosslinker. Data are shown for treatments with ChAbMT99/1, ChAbMT99/3, and human IgG at a concentration of 20 μg/mL.

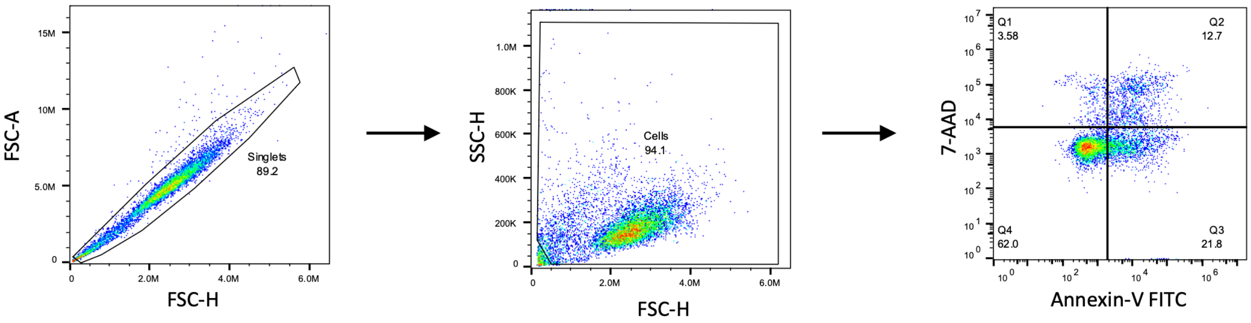

Figure S5. Gating strategy for apoptosis analysis of the Jurkat-derived spheroid model. Following spheroid dissociation into single-cell suspensions, singlet cells were identified based on forward scatter height (FSC-H) and forward scatter area (FSC-A). The singlet population was then gated using FSC-H and side scatter height (SSC-H) to identify the cell population for analysis. Apoptosis was subsequently evaluated by Annexin V–FITC and 7-AAD staining, and the percentages of live cells (Annexin V–FITC^{-}/7-AAD^{-}), early apoptotic cells (Annexin V–FITC^{+}/7-AAD^{-}), late apoptotic cells (Annexin V–FITC^{+}/7-AAD^{+}), and necrotic cells (Annexin V–FITC^{-}/7-AAD^{+}) were determined.

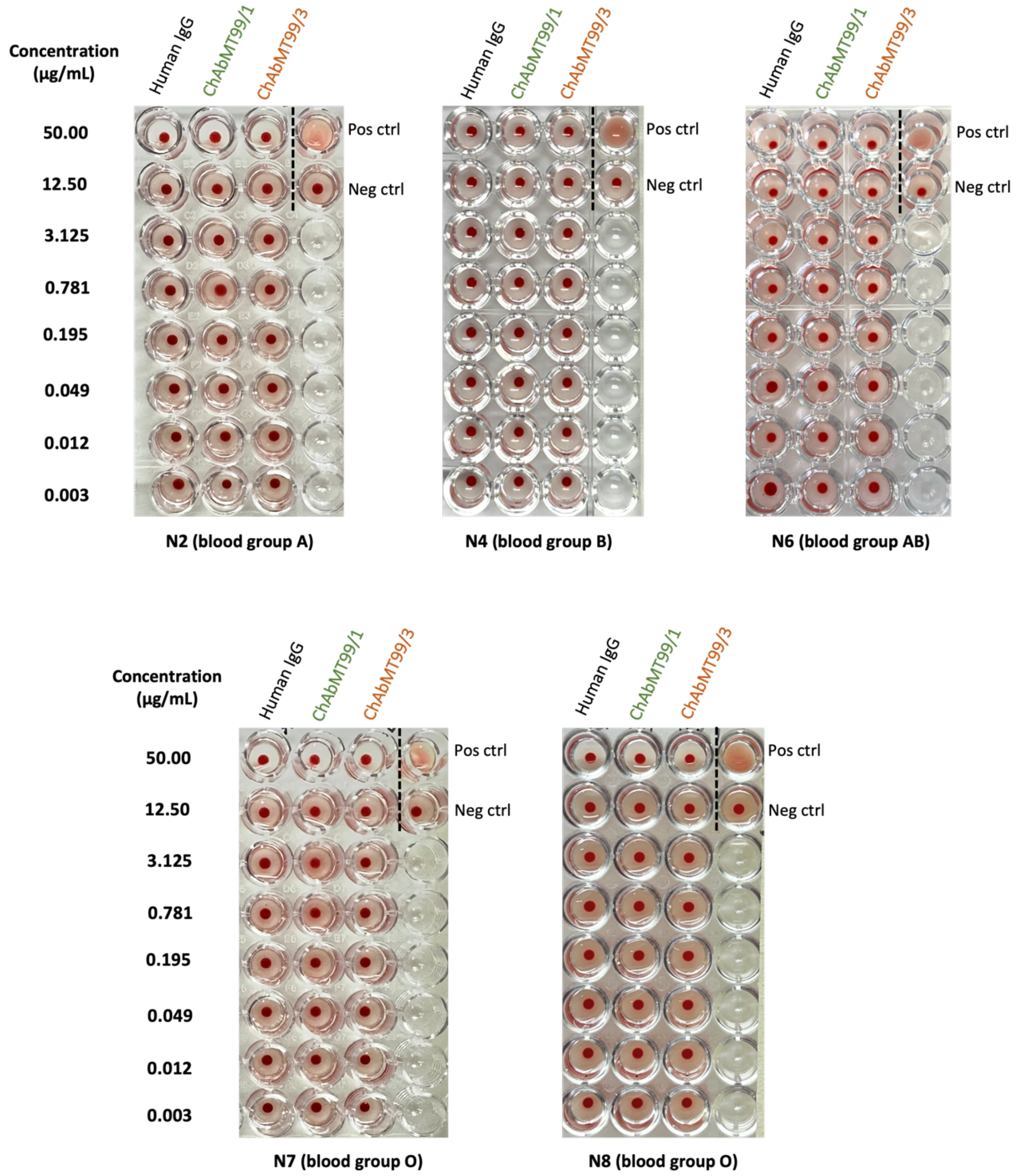

Figure S6. Hemagglutination results from five selected donors. Hemagglutination assays were performed by incubating a 2% RBC suspension with ChAbMT99/1, ChAbMT99/3, or an isotype-matched control antibody (human IgG) at various concentrations in U-bottom 96-well plates for 60 min. Anti-D IgM/IgG antibody was used as a positive control (Pos ctrl), whereas the negative control (Neg ctrl) consisted of RBCs incubated with PBS alone (no antibody).

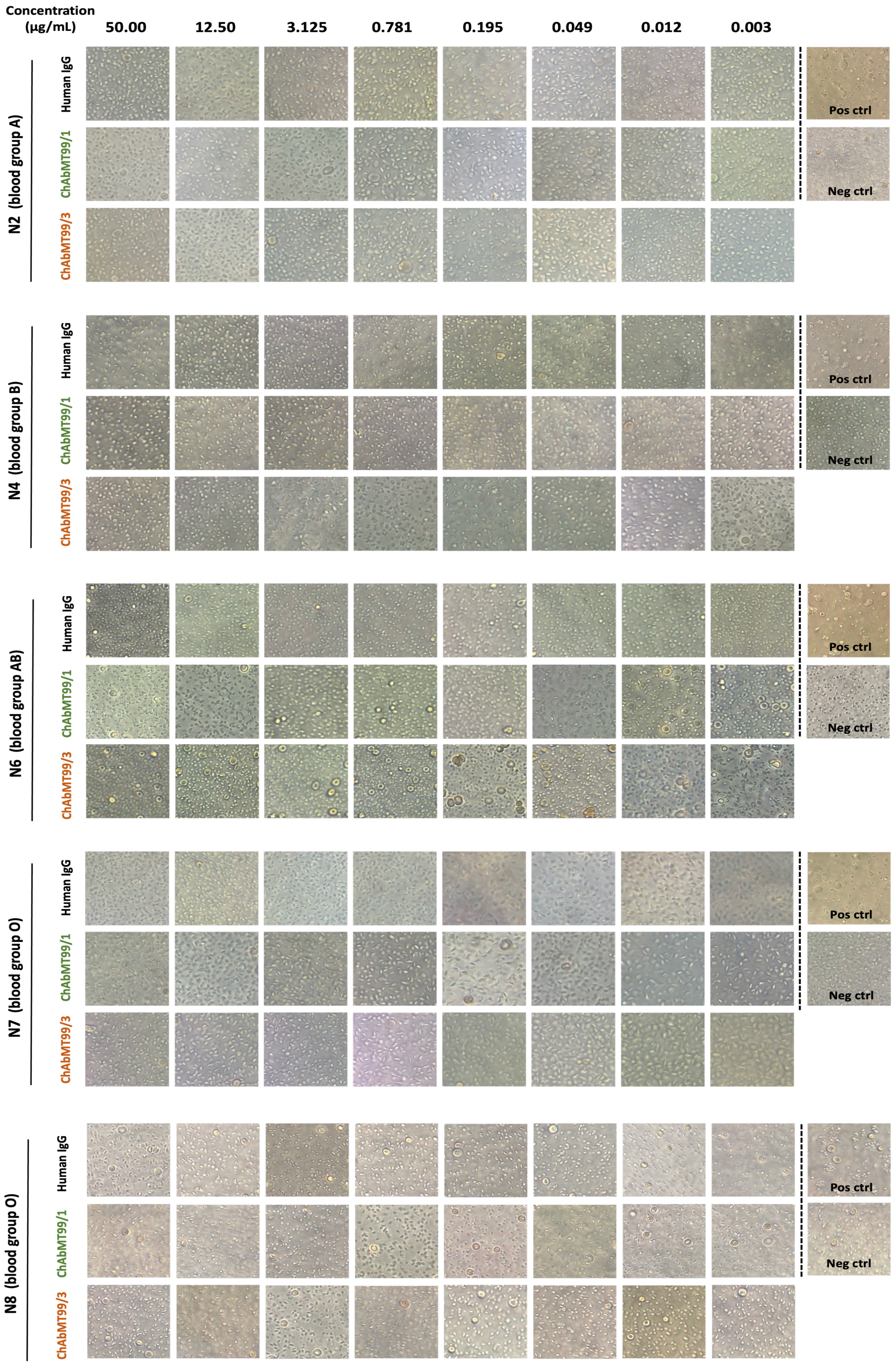
Concentration (µg/mL)
50.00
12.50
3.125
0.781
0.195
0.049
0.012
0.003
N2 (blood group A)
N4 (blood group B)
N6 (blood group AB)
N7 (blood group O)
N8 (blood group O)
Human IgG
ChAbMT99/1
ChAbMT99/3
Pos ctrl
Neg ctrl

Figure S7. Platelet aggregation results from five selected donors. Platelet-rich plasma (PRP) was incubated with ChAbMT99/1, ChAbMT99/3, or an isotype-matched control antibody (human IgG) at various concentrations in a flat-bottom 96-well plate for 30 min. Adenosine diphosphate (ADP) was used as a positive control (Pos ctrl; 30 min incubation), whereas the negative control (Neg ctrl) consisted of PRP incubated with PBS alone (no antibody; 30 min incubation).

Table S1. Production information for chimeric anti-CD99 antibodies.

Chimeric antibody clone	Concentration	Antibody yield	Culture supernatant volume for purification
ChAbMT99/1	3.42 mg/mL	10.07 mg	320 mL
ChAbMT99/3	0.584 mg/mL	2.6 mg	750 mL

Table S2. Comparison of area under the curve (AUC) values between ChAbMT99/1 and ChAbMT99/3, with exact *p*-values determined by the Wilcoxon matched-pairs signed-rank test.

Cell type	Exact *p*-value
Jurkat E6.1	0.3750
MOLT-4	0.2500
Lymphocytes	0.0625
Monocytes	0.0625

Table S3. Comparison of geometric mean fluorescence intensity (GeoMFI) values of RBCs and platelets using one-way ANOVA followed by Tukey's multiple comparisons test, with exact *p*-values and 95% confidence intervals (CI).

	hIgG vs ChAbMT99/1		hIgG vs ChAbMT99/3		ChAbMT99/1 vs ChAbMT99/3	
	Exact *p*-value	95% CI	Exact *p*-value	95% CI	Exact *p*-value	95% CI
**RBCs**	0.0388	-1431 to -33.93	0.0184	-1526 to -129.4	0.9368	-793.8 to 602.8
**Platelets**	0.0001	-2898 to -988.0	0.0007	-2626 to -715.7	0.7553	-682.7 to 1227

Table S4. Statistical analysis of the 2D apoptosis assay using one-way ANOVA followed by Tukey's multiple comparisons test, with exact *p*-values. (Without crosslinker conditions)

Cells	Concentration (µg/mL)	hIgG vs ChAbMT99/1	hIgG vs ChAbMT99/3	ChAbMT99/1 vs ChAbMT99/3
Jurkat E6.1	20.0	0.5635	0.9566	0.7238
	2.0	0.9740	0.9462	0.9946
	0.2	0.9849	0.3558	0.2921
MOLT-4	20.0	0.1609	0.9516	0.2351
	2.0	0.8681	0.9963	0.8278
	0.2	0.9915	0.5842	0.6544
PBMCs	20.0	0.1989	0.2687	0.9777
	2.0	0.5121	0.1502	0.6605
	0.2	0.9596	0.4759	0.6355

Table S5. Statistical analysis of the 2D apoptosis assay using one-way ANOVA followed by Tukey's multiple comparisons test, with exact *p*-values. (With crosslinker conditions)

Cells	Concentration (µg/mL)	hIgG vs ChAbMT99/1	hIgG vs ChAbMT99/3	ChAbMT99/1 vs ChAbMT99/3
Jurkat E6.1	20.0	0.0364	0.1844	0.4484
	2.0	0.0420	0.3199	0.3078
	0.2	0.1571	0.3671	0.7779
MOLT-4	20.0	0.0006	0.0035	0.1705
	2.0	0.0033	0.2154	0.0239
	0.2	0.0313	0.1682	0.4227
PBMCs	20.0	0.0002	0.0114	0.0873
	2.0	0.0578	0.0284	0.9160
	0.2	0.3514	0.3652	0.9995

Table S6. Statistical analysis of the 2D apoptosis assay using one-way ANOVA followed by Tukey's multiple comparisons test, with 95% confidence intervals (CI). (Without crosslinker conditions)

Cells	Concentration (μg/mL)	hIgG vs ChAbMT99/1	hIgG vs ChAbMT99/3	ChAbMT99/1 vs ChAbMT99/3
Jurkat E6.1	20.0	-12.41 to 5.983	-10.05 to 8.343	-6.836 to 11.56
	2.0	-5.752 to 4.986	-5.926 to 4.812	-5.542 to 5.196
	0.2	-5.175 to 5.768	-8.145 to 2.798	-8.442 to 2.502
MOLT-4	20.0	-8.308 to 1.474	-5.371 to 4.411	-1.954 to 7.828
	2.0	-4.353 to 3.107	-3.630 to 3.830	-3.007 to 4.453
	0.2	-2.901 to 2.675	-3.728 to 1.848	-3.615 to 1.961
PBMCs	20.0	-3.877 to 0.7131	-3.703 to 0.8871	-2.121 to 2.469
	2.0	-3.237 to 1.305	-3.989 to 0.5531	-3.023 to 1.519
	0.2	-2.232 to 1.816	-2.934 to 1.114	-2.726 to 1.322

Table S7. Statistical analysis of the 2D apoptosis assay using one-way ANOVA followed by Tukey's multiple comparisons test, with 95% confidence intervals (CI). (With crosslinker conditions)

Cells	Concentration (μg/mL)	hIgG vs ChAbMT99/1	hIgG vs ChAbMT99/3	ChAbMT99/1 vs ChAbMT99/3
Jurkat E6.1	20.0	-44.42 to -1.816	-35.44 to 7.168	-12.32 to 30.29
	2.0	-18.74 to -0.4275	-13.90 to 4.413	-4.316 to 14.00
	0.2	-8.669 to 1.502	-7.526 to 2.646	-3.942 to 6.229
MOLT-4	20.0	-51.66 to -22.10	-41.55 to -11.99	-4.675 to 24.89
	2.0	-30.80 to -8.989	-17.70 to 4.107	2.193 to 24.00
	0.2	-7.231 to -0.4295	-5.737 to 1.064	-1.907 to 4.894
PBMCs	20.0	-7.797 to -2.907	-5.647 to -0.7567	-0.2953 to 4.595
	2.0	-4.303 to 0.06750	-4.631 to -0.2605	-2.513 to 1.857
	0.2	-2.815 to 0.8393	-2.795 to 0.8593	-1.807 to 1.847

Table S8. Statistical analysis of the Jurkat-derived 3D spheroid apoptosis assay using one-way ANOVA followed by Tukey's multiple comparisons test, with exact *p*-values and 95% confidence intervals (CI).

	**Exact *p*-value**	**95% CI**
hIgG vs ChAbMT99/1	0.0059	-43.21 to -10.30
hIgG vs ChAbMT99/3	0.0148	-38.52 to -5.603
ChAbMT99/1 vs ChAbMT99/3	0.6738	-11.76 to 21.15

Table S9. Minimum Information about a Flow Cytometry Experiment (MIFlowCyt) reporting information.

Model: Accuri C6 plus or Accuri C6 flow cytometer (BD Biosciences, San Jose, California, USA)

Configuration: standard dual-laser configuration (488 nm blue and 640 nm red lasers), two light scatter detectors (forward scatter and side scatter), and four fluorescence detectors (FL1 (533/30 nm), FL2 (585/40 nm), FL3 (670 nm LP) for the blue laser; FL4 (675/25 nm) for the red laser)

Experiment	Flow sample		Data analysis		Settings	
	Cells	Treatment	Gating	Compensation	Event counts	FSC threshold value
Binding reactivity	Jurkat E6.1<br>MOLT-4<br>PBMCs	Human IgG<br>ChAbMT99/1<br>ChAbMT99/3	Figure S3a-b	No compensation	10,000 cells in FSC/SSC gating	80,000
	RBCs<br>Platelets	Human IgG<br>ChAbMT99/1<br>ChAbMT99/3	Figure S3c	Software compensation [#]<br><br>(Correct FL2-H by subtracting FL1-H 8.2%)	25,000-30,000 cells in FSC/SSC gating	80,000
Apoptosis<br>(2D culture)	Jurkat E6.1<br>MOLT-4<br>PBMCs	Human IgG<br>ChAbMT99/1<br>ChAbMT99/3	Figure S4a	Software compensation*<br><br>(Correct FL3-H by subtracting FL1-H 4%, Correct FL2-H by subtracting FL1-H 11.7%, Correct FL3-H by subtracting FL2-H 29.06%, Correct FL1-H by subtracting FL2-H 2.27%)	10,000 cells in FSC/SSC gating	80,000

[#] Compensation was performed using single-stained control and applied during data acquisition using BD Accuri C6 software.

*Compensation was performed using single-stained control and applied during data acquisition using BD Accuri C6 plus software.

Table S9. Minimum Information about a Flow Cytometry Experiment (MIFlowCyt) reporting information (cont.).

Experiment	Flow sample		Data analysis		Settings	
	Cells	Treatment	Gating	Compensation	Event counts	FSC threshold value
Apoptosis (Jurkat-derived 3D spheroids)	Jurkat E6.1	Human IgG<br>ChAbMT99/1<br>ChAbMT99/3	Figure S5	Software compensation*<br><br>(Correct FL3-H by subtracting FL1-H 3.6%, Correct FL2-H by subtracting FL1-H 12.23%, Correct FL3-H by subtracting FL2-H 32.28%, Correct FL1-H by subtracting FL2-H 2.17%)	9,000-10,000 cells in FSC/SSC gating	80,000

Compensation was performed using single-stained control and applied during data acquisition using BD Accuri C6 software.

*Compensation was performed using single-stained control and applied during data acquisition using BD Accuri C6 plus software.
